# Supplementary material for: Targeting operational regimes of interest in recurrent neural networks
Source: PLoS Comput Biol. 2023 May 15;19(5):e1011097. doi: 10.1371/journal.pcbi.1011097 (PMC10212141; doi:10.1371/journal.pcbi.1011097)
Supplement: S1 Text — (ZIP) [file pcbi.1011097.s003.zip › S1_Text.pdf]

S1 Text

Extraction of experimentally reported network parameters

We use the Cell Type and the Synaptic Physiology databases from the Allen institute [1] to derive biologically plausible network parameters based on the data collected for layer 2/3 of the mouse visual cortex. Nevertheless, our analyses are not restricted to this brain region, as our framework is applicable to any cortical network. It should be noted that the reported values of network parameters vary largely between experimental reports [2, 3, 4, 5, 6]. Furthermore, detailed network simulations using state-of-the-art experimentally measured parameters require optimization of all recurrent connection weights in order to generate realistic spiking activity [7, 8]. Therefore, we use the parameters for mouse V1 extracted from the Allen institute database [1] as a starting point from which we can explore the range of biologically plausible network connectivity.

We use the Cell Type database [1], to obtain the membrane time constant (τ) as well as the membrane resting and threshold potentials (V_R and Θ), for E and I neurons. The data has been obtained through whole cell patch clamp recording. We use the Synaptic Physiology database [1] to derive the synaptic strength and the probability of connection (j_{XY} and p_{XY}) between E and I neurons. The data has been obtained with octopatching, the simultaneous patch-clamp recording of up to eight neurons. The neurons whose dendrite type is classified as *spiny* constitute the E population. The I population consists of all neurons classified as VIP, PV and Sst.

The reported neuronal properties (τ, V_R and Θ) were obtained by averaging the recorded values from 66 E neurons and 94 I neurons. The probability of connection (p_{XY}) is the fraction of connected pairs with respect to all probed pairs, where 80 EE, 150 EI, 160 IE and 607 II cell pairs were probed, Table A. Finally, we derive the connection strength (j_{XY}) using the peak amplitude (A_{PSP}), the rise time (t_R) and the decay time constant (τ_D) of the postsynaptic potential. These parameters are measured over 66 EE, 46 EI, 3 IE and 29 II connected cell pairs. The mean-field analysis of the network at equilibrium does not depend on the dynamical properties of the synaptic transmission (i.e. the shape of the PSP profile). Instead, the mean-field strength of the synaptic connection is the overall depolarization caused by a single presynaptic spike and it is characterized by a single value j_{XY}, regardless of the dynamics. It is given by the time integral of the synaptic current elicited by one presynaptic spike. We assume that the profile of the postsynaptic membrane potential following a presynaptic spike is a linear increase followed by an exponential decay and that the recorded postsynaptic neuron was at resting potential before receiving its input.

$$PSP(t) = \begin{cases} V_R + \frac{A_{PSP}}{t_R}\, t & \text{if } t < t_R \\ V_R + A_{PSP}\, e^{-\frac{t-t_R}{\tau_D}} & \text{if } t > t_R. \end{cases}$$

Using the LIF equation (Eq 8) for a single pre-synaptic spike ($\int I dt = j_{XY}$) yields

$$j_{XY} = \int_0^\infty \left(\frac{dV}{dt} + \frac{V}{\tau} \right) dt = (V_R - V_R) + \frac{\int_0^\infty V\, dt}{\tau} = A_{PSP} \frac{t_R/2 + \tau_D}{\tau}.$$

The synaptic strength is then normalized by the experimentally recorded value of $\Theta - V_R$ [1], so that V_R and Θ can be set to 0 and 1, respectively.

By default, we assume that both populations receive the same external input ($r = 1$). Finally, we assume the input noise σ_{ext} to be 3 mV/$\sqrt{s}$, which leads to a power law exponent in the F-I curve close to 3, similar to the values reported in [9, 10] (Table D - E and I populations).

The neuronal parameters used in LIF spiking network simulations are presented in Table A. Fig 3A is generated with the mouse V1 parameters we extracted from the Allen institute database [1]. We explore the range of biologically plausible network connectivity parameters by modifying the individual connection strengths J_{XY} while keeping them within the range delimited by the lowest and largest experimentally reported connectivity values in mouse V1 ($0.5 \le J_{XY} \le 25$). The connectivity parameters used in all network simulations are presented in Table B.

N_E	3000	
N_I	1000	
p_{EE}	6.5	%
p_{EI}	20	%
p_{IE}	27.5	%
p_{II}	10	%
V_r	0	mV
θ	1	mV
τ_E	20	ms
τ_I	10	ms
σ_{ext}	3	mV/$\sqrt{s}$
r	1	

Table A: Spiking network parameters used in all figures by default. Parameters deviating from the default ones are specified in figures' panels and captions. For derivation, see **section Extraction of experimentally reported network parameters**.

	j_{EE}	j_{EI}	j_{IE}	j_{II}	J_{EE}	J_{EI}	J_{IE}	J_{II}
Fig 2<br>Fig 6A-B<br>S1 FigA	10.3	60	7.27	10	2	12	6	1
Mouse V1<br>Fig 3A<br>S1 FigB	3.59	68.1	28.7	108	0.672	13.2	23.7	11.8
Fig 3B	25.4	68.1	28.7	108	4.75	13.2	23.7	11.8
Fig 4<br>Fig 5A<br>Fig 6C-D<br>S2 Fig	25.6	50	8.49	110	5	10	7	11
Fig 5B<br>Fig 6E-F	6.41	-5	1.21	-12.5	1.25	1	1	1.25

Table B: Network connectivity parameters used in all figures. The synaptic weights j_{XY} correspond to the strength of a single spike in spiking networks and are given in μV. The connection strength at the population level J_{XY}, used in mean field solutions, are given in mV (Eq 13). For Fig 6 and S2 Fig, synaptic weights are N-dependent since the $1/\sqrt{N}$ scaling is applied; the given values correspond to $N = 4000$. See **section Extraction of experimentally reported network parameters**.

It should be noted that the magnitude of the recurrent connection strengths we obtain is low enough to avoid instability. At the same time, the recurrent connections are not negligible as the response of the network to input differs from the purely feedforward scenario, equivalent to disconnected neurons (F-I curves shown in Fig 1B).

Finally, for some spiking network simulations we used exponential synapses with randomly distributed synaptic delays to avoid synchronization (Section LIF spiking network simulation). In those cases, the values of the synaptic time constant τ_S and the delay D are included only to stabilize the network around the steady state. The particular parameter values should not substantially affect the mean-field solution as we operate at steady state. While the use of exponential synapses can lead to some changes in the properties of the recurrent noise [11, 12], the contribution of recurrent noise is negligible compared to external noise (Section Mapping LIF network-SSN) and is not considered in the SSN framework. The values of τ_S and D are provided in Table **C**.

	τ_S	D	Affected Synapses
Fig 4<br>Fig 6C<br>Fig 6E<br>S2 Fig	10	0 - 100	EE,II

Table C: Synaptic parameters used to prevent synchronization. The exponential synapse time constant τ_S and the synaptic delay D are given in ms. The synaptic delay for each synapse is drawn randomly from a uniform distribution in the given range. The EI and IE synapses are not affected and use delta-synapses without delay. In all figures not listed here, the LIF simulations were performed with the default delta-synapses without delay.

SSN parameters	a	b	n
Excitatory population	1.08×10^{-4}	-11.1	3.08
Inhibitory population	2.21×10^{-6}	4.8	3.82
Fig 1 ($\sigma = 1$ mV/$\sqrt{\text{s}}, \tau = 20$ ms)	3.91×10^{-2}	31.6	2.15
Fig 1 ($\sigma = 3$ mV/$\sqrt{\text{s}}, \tau = 15$ ms)	3.23×10^{-5}	-6.4	3.31
Fig 1 ($\sigma = 5$ mV/$\sqrt{\text{s}}, \tau = 10$ ms)	4.90×10^{-8}	-63.0	4.20
Fig 4B ($\sigma = 3$ mV/$\sqrt{\text{s}}, \tau = 18$ ms)	7.89×10^{-5}	-9.5	3.14
Fig 4B ($\sigma = 3$ mV/$\sqrt{\text{s}}, \tau = 22$ ms)	1.60×10^{-4}	-12.1	3.01

Table D: SSN parameters used in all figures. The parameters a, b, and n are based on the power-law fit of the LIF F-I curve (Eq 1), with the input μ in mV/s and the firing rate ν in Hz.

Deriving biologically plausible neural network size for circuit simulations

In our framework, we assume that a network consists of populations of neurons which share a similar external input and preferentially connect together with a homogeneous connection probability and strength. All connections originating from outside this circuit are considered to be feedforward input (see schematic Fig 1A, bottom). In biological circuits, it is difficult to determine what can constitute a single network since the brain exhibits a high degree of complexity and does not consist of well-separated circuits. Nonetheless, the analysis of cortical regions in which projection columns can be anatomically identified leads to a consistent order of magnitude for network sizes. Here we set the typical functional network to consist of 3000 E and 1000 I neurons. We present the corresponding citations below.

In the primary visual cortex of mice, it is especially challenging to define a local network size since the cortical map is unstructured, meaning that neurons which share the same receptive field do not co-localize [13]. However, this does not mean that the concept of homogeneous network cannot be applied to mouse V1, as neurons which share the same receptive field preferentially connect together [2]. Since local networks within mouse V1 cannot be defined based on spatial anatomy, we used other brain regions with anatomically-defined networks to define a reference point.

In mouse somatosensory cortex, distinct neuroanatomical structures known as barrels each receive the sensory input from a corresponding whisker [14]. These structures are perfect candidates to define the typical scale of a homogeneous network. Their diameter ranges from 100 to 400 μm, with a thickness in layer 4 of 100 μm [14]. Using the neuronal density observed in [15], the number of neurons in these structures ranges from 140 to 2200. In the rat barrel cortex, the number of neurons in each layer of multiple projection columns has specifically been counted [16], and is on the order of $N = 4000$ in layer 4 and $N = 6000$ in layer 2/3.

In primates, the primary visual cortex of macaques has been studied extensively. Unlike rodents, the cortical organization of macaque V1 shows a columnar structure, both for eye dominance and orientation preference [17]. Within a range of orientation preference of 10°, such columns are slab-shaped, with a size of 30 μm by 0.5 to 1mm [18]. With the thickness of layer 2 and layer 3 being respectively 225 μm and 310 μm [19], and a cell density of 1.3×10^5 neurons/mm^3 [20], we can deduce that the number of neurons in layer 2/3 in

such columns is in the range of 1000 to 2000.

In summary, it appears that across species and cortical regions, we can define functional networks with sizes ranging from hundreds to a few thousands of neurons. In this work, we choose a network size of 4000 because it corresponds to the value reported in [16], which is the only study where the neurons in a cortical column were directly counted. In this context, we can use anatomical analyses of neurons in the same brain region to determine the fraction of excitatory and inhibitory neurons in a network [21, 22], which leads to a E/I ratio of 3.5 in layer 2/3. This corresponds to 3110 E neurons and 890 I neurons, which we round to 3000 and 1000 respectively.

Finally, we verify that the network size we assume (i.e. 4000 neurons) is plausible for mouse V1, since we could not use a spatial anatomy feature to define a network. We use the reported probability profile (p) for E→E connections as a function of distance for mouse V1 layer 2/3, Fig 4B in [23] (near 20% for nearby pairs, going down to 0% for neuron pairs 150μm apart). Following this observation, the number of excitatory synapses to an excitatory neuron can be obtained through a 3D spatial integration of this connection probability profile: $n_c = \int\int\int p(\sqrt{x^2+y^2+z^2})\eta dxdydz$, where η is the density of excitatory neurons. Over an infinite 3D space, we obtain the total number of E→E connections: $n_c = 8.7 \times 10^5 \eta$. Using a neuronal density of 1.64×10^{-4} neurons/μm^3 [24], and assuming a E/I ratio of 3.5, we obtain $n_c \approx$111 connections. The result of this rough calculation is in the same order of magnitude as the 195 connections we obtain with 4000 neurons and a probability of connection of 6.5% (as described in [1]), which suggests that this network size is a valid approximation for mouse V1 as well. It should be noted that the network we define does not constitute a single block of cortex due to the salt-and-pepper organization of this brain region, but consists instead of distant neurons which receive the same external input and are homogeneously connected within the network.

Derivation of parameter conditions for specific computational regimes

Balanced state framework

Here, we provide for completeness the solutions of the balanced state framework derived previously [25] which we used as a reference to study the convergence to the balanced state. In this framework, the mean input to each population vanishes as the number of neurons in the network increases, $\mu_E \approx \mu_I \approx 0$ and the equations for μ_E and μ_I in Eq 14 can be simplified to

$$\begin{aligned}\mu_{\text{ext}} =& J_{EI}\nu_I - J_{EE}\nu_E \\ r \times \mu_{\text{ext}} =& J_{II}\nu_I - J_{IE}\nu_E.\end{aligned}$$

The solution of the balanced state equations reads

$$\begin{aligned}\nu_E =& \frac{rJ_{EI} - J_{II}}{J_{EE}J_{II} - J_{EI}J_{IE}}\mu_{\text{ext}} \\ \nu_I =& \frac{rJ_{EE} - J_{IE}}{J_{EE}J_{II} - J_{EI}J_{IE}}\mu_{\text{ext}}.\end{aligned} \tag{S1}$$

The balanced state solution is only valid if both ν_E and ν_I are positive for positive input, which corresponds to the following condition on connectivity

$$\begin{cases} \dfrac{rJ_{EI} - J_{II}}{J_{EE}J_{II} - J_{EI}J_{IE}} > 0 \\ \\ \dfrac{rJ_{EE} - J_{IE}}{J_{EE}J_{II} - J_{EI}J_{IE}} > 0. \end{cases} \tag{S2}$$

Stability of the balanced state

Within the balanced state framework, we assume that the change in firing rate of each population is a function of the excess input it receives.

$$\frac{d\nu_E}{dt} = f(\mu_E)$$
$$\frac{d\nu_I}{dt} = g(\mu_I).$$

The firing rate of a population is at the steady state when its total input is balanced ($f(0) = 0$ or $g(0) = 0$). By linearising around a steady state, we get

$$\nu_E = \nu_E^0 + \delta\nu_E$$
$$\nu_I = \nu_I^0 + \delta\nu_I$$

$$\dot{\nu_E^0} + \dot{\delta\nu_E} = f(\mu_E^0 + \delta\mu_E)$$
$$\dot{\nu_I^0} + \dot{\delta\nu_I} = g(\mu_I^0 + \delta\mu_I)$$

$$\dot{\delta\nu_E} = f'(\mu_E^0)\delta\mu_E = f'(\mu_E^0)(J_{EE}\delta\nu_E - J_{EI}\delta\nu_I)$$
$$\dot{\delta\nu_I} = g'(\mu_I^0)\delta\mu_I = g'(\mu_I^0)(J_{IE}\delta\nu_E - J_{II}\delta\nu_I)$$

This can be rewritten as

$$\begin{bmatrix} \dot{\delta\nu_E} \\ \dot{\delta\nu_I} \end{bmatrix} = \begin{bmatrix} f' & 0 \\ 0 & g' \end{bmatrix} \begin{bmatrix} J_{EE} & -J_{EI} \\ J_{IE} & -J_{II} \end{bmatrix} \begin{bmatrix} \delta\nu_E \\ \delta\nu_I \end{bmatrix}.$$

Where f' and g' are positive (excess input drives the firing rate up), the state (ν_E^0, ν_I^0) is stable if the two eigenvalues of the Jacobian matrix

$$\begin{bmatrix} f'J_{EE} & -f'J_{EI} \\ g'J_{IE} & -g'J_{II} \end{bmatrix}$$

have negative real parts.

The eigenvalues λ_1 and λ_2 are roots of the polynomial

$$\lambda^2 - \lambda(f'J_{EE} - g'J_{II}) + f'g'(J_{EI}J_{IE} - J_{EE}J_{II})$$

or

$$\lambda_1, \lambda_2 = \left(\frac{f'J_{EE} - g'J_{II}}{2}\right)\left(1 \pm \sqrt{1 - \frac{4f'g'\det J}{(f'J_{EE} - g'J_{II})^2}}\right).$$

The steady state is stable $\iff$

$$\begin{cases} f'J_{EE} - g'J_{II} < 0 \\ \det J > 0 \end{cases} \tag{S3}$$

The first condition requires that the response of the inhibitory population (g') is fast and strong enough to prevent a runaway excitatory feedback loop. We do not use this condition here because it depends on the

dynamic properties of the network (f and g functions), which are beyond the scope of this work. However, the second condition constrains the connectivity matrix such that $J_{EI}J_{IE} - J_{EE}J_{II} > 0$ [26].

The condition on the existence of a non-negative balanced state (Eq S2) can be combined with the stability condition on connectivity (Eq S3) to delineate the parameter range where a balanced state limit exists and is stable [25, 26]:

$$\begin{cases} 0 < r < \min(\frac{J_{II}}{J_{EI}}, \frac{J_{IE}}{J_{EE}}) \\ \det J > 0 \end{cases} \tag{S4}$$

We use Eq S4 to visualize the parameter range where a balanced state exists in Fig 5 and S1 Fig.

Condition on supersaturation

Supersaturation is characterized by a decrease in the excitatory firing rate with increasing external input: $\frac{d\nu_E}{d\mu_{\text{ext}}} < 0$.

Linearizing the system around a fixed point, leads to the relation between input and firing rate at the steady state [27]:

$$\begin{bmatrix} \delta\nu_E \\ \delta\nu_I \end{bmatrix} = \left(\begin{bmatrix} 1 & 0 \\ 0 & 1 \end{bmatrix} - \underbrace{\begin{bmatrix} f'_E & 0 \\ 0 & f'_I \end{bmatrix}}_{=F} \underbrace{\begin{bmatrix} J_{EE} & -J_{EI} \\ J_{IE} & -J_{II} \end{bmatrix}}_{=J} \right)^{-1} \left(\begin{bmatrix} f'_E & 0 \\ 0 & f'_I \end{bmatrix} \begin{bmatrix} \delta\mu_{\text{extE}} \\ \delta\mu_{\text{extI}} \end{bmatrix} \right).$$

Where $\delta\nu$ and $\delta\mu_{\text{ext}}$ are the firing rates and external inputs linearized around a steady state. The functions f_E and f_I are the input-firing rate transfer functions of the two populations. The functions f'_E and f'_I are the derivatives with respect to input, calculated at the fixed point. The effect of a change of external input yields:

$$\begin{bmatrix} \delta\nu_E \\ \delta\nu_I \end{bmatrix} = \frac{\begin{bmatrix} f'_E\,(1 + f'_I J_{II}) & -f'_E f'_I J_{EI} \\ f'_E f'_I J_{IE} & f'_I\,(1 - f'_E J_{EE}) \end{bmatrix}}{\left|I - FJ\right|} \begin{bmatrix} \delta\mu_{\text{extE}} \\ \delta\mu_{\text{extI}} \end{bmatrix}. \tag{S5}$$

In particular, the effect of external input on the excitatory firing rate yields

$$\frac{d\nu_E}{d\mu_{\text{ext}}} = \frac{f'_E\,(1 + f'_I\,(J_{II} - rJ_{EI}))}{\left|I - FJ\right|}$$

As shown in [27], $\left|I - FJ\right|$ must be positive for the fixed point to be stable. Furthermore, f'_I and f'_E are assumed to be positive, meaning that the F-I curves are monotonically increasing. This leads to the following condition for supersaturation:

$$f'_I\,(J_{II} - rJ_{EI}) < -1.$$

Which is only possible if $r > \frac{J_{II}}{J_{EI}}$. In the SSN, the transfer function is a power law (Eq 1). Its derivative is then $f'_X = n_X a_X^{\frac{1}{n_X}} \nu_X^{\frac{n_X-1}{n_X}}$. This leads to the condition that supersaturation occurs when the fixed point inhibitory firing rate is sufficiently high [28]:

$$\nu_I > \sqrt[n_I-1]{\frac{1}{a_I\,(n_I\,(rJ_{EI} - J_{II}))^{n_I}}} \tag{S6}$$

For completeness, it is worth mentioning that there exists one particular edge-case where $r > \frac{J_{II}}{J_{EI}}$ cannot not lead to $\frac{d\nu_E}{d\mu_{\text{ext}}} < 0$. This occurs when the only stable state of the system is such that $\nu_E = 0$ regardless of μ_{ext}. This is discussed in **section Effect of J_{EE} on the occurence of ISN** and requires $rb_E - b_I > 0$. Since these cases correspond to situations where recurrent inhibition is strong enough to prevent any excitatory activity, we consider them to be supersaturating.

Modulation of E firing rate peak in supersaturating activity regime

Here, we explain how we modified the height of the E firing rate peak in Fig 2B. We begin by analyzing how the value of maximal E firing rate depends on SSN parameters for supersaturating networks. A characterizing property of the maximal E firing rate ν_E is that it satisfies $\frac{d\nu_E}{d\mu_{\text{ext}}} = 0$. As shown previously [28], this occurs for $\nu_I = (a_I^{\frac{1}{n_I}} n_I(J_{EI}r - J_{II}))^{-\frac{n_I}{n_I-1}}$, where $J_{EI}r - J_{II}$ must be positive. Since ν_I and ν_E are both positive, we can remove the $(\cdot)_+$ operator in the SSN equations and apply the inverse power law exponents to both sides to express μ_{ext} in Eq 3

$$a_E^{-\frac{1}{n_E}} \nu_E^{\frac{1}{n_E}} - \big(J_{EE}\nu_E - J_{EI}\nu_I - b_E\big) = \mu_{\text{ext}}$$
$$r^{-1} a_I^{-\frac{1}{n_I}} \nu_I^{\frac{1}{n_I}} - r^{-1}\big(J_{IE}\nu_E - J_{II}\nu_I - b_I\big) = \mu_{\text{ext}}.$$

We combine two above equations to obtain

$$a_E^{-\frac{1}{n_E}} \nu_E^{\frac{1}{n_E}} + (\frac{J_{IE}}{r} - J_{EE})\nu_E = -(J_{EI} - r^{-1}J_{II})\nu_I + r^{-1}a_I^{-\frac{1}{n_I}} \nu_I^{\frac{1}{n_I}} - b_E + \frac{b_I}{r}. \tag{S7}$$

Substituting $\nu_I = (a_I^{\frac{1}{n_I}} n_I(J_{EI}r - J_{II}))^{-\frac{n_I}{n_I-1}}$ in the above equation, we obtain

$$a_E^{-\frac{1}{n_E}} \nu_E^{\frac{1}{n_E}} + (\frac{J_{IE}}{r} - J_{EE})\nu_E = \frac{1}{r}(a_I n_I(rJ_{EI} - J_{II}))^{-\frac{1}{n_I-1}}(1 - \frac{1}{n_I}) - b_E + \frac{b_I}{r}. \tag{S8}$$

The solution of Eq S8 corresponds to the maximal E firing rate in the supersaturating activity regime. To increase the E firing rate peak, we modified r, J_{IE}, and J_{EI}. Specifically, we decreased r and modified J_{IE} and J_{EI} such that the terms J_{IE}/r and $J_{EI}r$ remained constant. If $J_{IE}/r - J_{EE}$ is positive, the left-hand side of the equation is a monotonically increasing function of ν_E. As $1/r$ increases, the right side of Eq S8 moves upward and the corresponding ν_E on the left side must increase as well. In this way the unique solution ν_E of Eq S8 - the maximal E firing rate - increases as $1/r$ increases. The method demonstrated here assumes that the left hand side of Eq S8 is an increasing function of ν_E. This is the case if $J_{IE}/r - J_{EE}$ is positive (as in Fig 2), and the peak of the excitatory activity can be increased infinitely. On the other hand, if $J_{IE}/r - J_{EE}$ is negative, the peak of supersaturation is bounded. In particular, for supersaturating networks ($J_{II}/r - J_{EI} < 0$) for which $\det J$ is negative, $J_{IE}/r - J_{EE}$ is negative. The peak of supersaturation is therefore bounded, and decreasing r can lead to an unstable situation where no steady state exist (as illustrated in Fig 5B).

Condition on the paradoxical effect

The paradoxical effect [29, 27, 30] is characterized by a decrease of the I firing rate, as the external input to the I population is increased: $\frac{d\nu_I}{d\mu_{\text{extI}}} < 0$. Here again, the effect of a change in external input is given by Eq S5 where $\lvert I - FJ \rvert$ and f'_E must be positive. In this case, $\frac{d\nu_I}{d\mu_{\text{extI}}}$ can only be negative if

$$f'_E J_{EE} > 1.$$

This condition is equivalent to the condition for the instability of the excitatory subnetwork [27]. The paradoxical effect is therefore a feature of the inhibition stabilized network (ISN) as it occurs when the activity of the E population is only made stable thanks to the suppression from the I population. Using the SSN transfer function (Eq 1), the condition on the paradoxical effect leads to Eq 7.

Effect of J_{EE} on the occurence of ISN

Since the network is inhibition-stabilized for fixed points which satisfy Eq 7, it seems that any network can be in the ISN state, granted that J_{EE} is sufficiently strong. Here we ask whether there is any counterexample to this. Are there networks which cannot enter ISN, no matter how high J_{EE} is?

From Eq S7, we impose that $\nu_E = (a_E n_E^{n_E} J_{EE}^{n_E})^{-\frac{1}{n_E-1}}$, which corresponds to the onset of inhibition-stabilization to get

$$\left(\frac{\nu_I}{a_I}\right)^{\frac{1}{n_I}} - (rJ_{EI} - J_{II})\,\nu_I = \frac{1}{(a_E n_E J_{EE})^{\frac{1}{n_E-1}}}\left(r - \frac{r}{n_E} + \frac{J_{IE}}{n_E J_{EE}}\right) + rb_E - b_I. \qquad \text{(S9)}$$

This equation can be seen as the crossing of two functions where the left hand side f is a function of the I activity ν_I and the right-hand side g is independent of ν_I but varies with J_{EE}

$$f(\nu_I) = g(J_{EE}).$$

If there is a value of ν_I which satisfies this equation, the network has a fixed point such that ν_E is at the onset of ISN. We note from Eq S7 that

$$\frac{df}{d\nu_E} > 0 \iff \nu_E < a_E\left(a_E n_E\left(J_{EE} - \frac{J_{IE}}{r}\right)\right)^{-\frac{n_E}{n_E-1}}.$$

Since the value of ν_E at which we operate in Eq S9 is smaller than this (for $J_{IE} \neq 0$), the network cannot be in the ISN if f and g do not intersect and f remains below g.

The f function starts at 0 for $\nu_I = 0$ and is either monotonously increasing (if $J_{II} > rJ_{EI}$) and tends to ∞, or it reaches a maximum and then tends to $-\infty$ (if $rJ_{EI} > J_{II}$). The second case corresponds to supersaturation, where high values of ν_I can be sustained with low recurrent excitation. Whatever the parameters, f first rises and always has at least some positive values.

The g function can be represented as a polynomial

$$g(X) = \frac{J_{IE}}{n_E\,(a_E n_E)^{\frac{1}{n_E-1}}} X^{n_E} + \frac{r\,(n_E-1)}{(a_E n_E)^{\frac{1}{n_E-1}}} X + rb_E - b_I.$$

Where $X = J_{EE}^{-\frac{1}{n_E-1}}$. Since the coefficients in front of X^{n_E} and X are both positive and X is necessarily positive (because J_{EE} is positive), g can take all values larger than $rb_E - b_I$. This means that, with J_{EE} as a free parameter, equation Eq S9 will have a solution if there is any ν_I such that $f(\nu_I) > rb_E - b_I$.

For values of b_E and b_I such that $rb_E - b_I < 0$, the network can always enter an inhibition-stabilized state by tuning J_{EE}. Interestingly, this is always the case with the values of b_E and b_I we obtained from fitting the F-I curve (See Table D) because experimentally reported neuronal parameters [1] are such that $\tau_E > \tau_I$. Similarly, in networks which do not satisfy the supersaturation condition ($J_{II} > rJ_{EI}$), g always crosses f.

On the other hand, in networks for which f has a maximum ($J_{II} > rJ_{IE}$), if $rb_E - b_I$ is higher than this maximum, the functions f and g never cross regardless of the value of J_{EE}. In this scenario, the network can never reach a steady state where it is inhibition-stabilized. These cases correspond to situations where the E activity is always suppressed and there is no stable steady state with $\nu_E > 0$. If J_{EE} is large, another unstable steady state will exist (for $\det J < 0$, see **section Parity of solutions** below), and if the system is perturbed enough to reach it the E activity will enter an unlimited feedback loop which leads to always increasing activity. These systems cannot be in the ISN regardless of J_{EE} because the E activity is either entirely silent or cannot be stabilized by inhibition.

Condition on multiplicity of solutions

As shown in [31], the two-dimensional SSN equation (Eq 3) can be rewritten as a single characteristic function $\mathcal{F}$, where the steady states of the system correspond to zeros of $\mathcal{F}$.

$$\mathcal{F}(\mu_E) = a_E J_{EE} \left(\mu_E - b_E\right)_+^{n_E} - a_I J_{EI} \left(\mu_I(\mu_E) - b_I\right)_+^{n_I} - \mu_E + \mu_{\text{ext}}.$$

Where μ_I is a function of μ_E : $\mu_I(\mu_E) = \frac{a_E \det J}{J_{EI}} \left(\mu_E - b_E\right)_+^{n_E} + \frac{J_{II}}{J_{EI}}\mu_E + \mu_{\text{ext}} \left(r - \frac{J_{II}}{J_{EI}}\right)$. The first term of $\mathcal{F}$ is zero if the E population is silenced ($\nu_E = 0$), and the second one if the I population is silenced ($\nu_I = 0$). For any value μ_E which satisfies $\mathcal{F}(\mu_E) = 0$, the corresponding excitatory firing rate is given by the power law F-I function Eq 1.

The number of zero crossings of the function $\mathcal{F}$ corresponds to the number of fixed points of the system. Since $\mathcal{F}$ is a continuous function, its number of zero crossings only changes when two solutions merge into one or when one solution splits into two. This corresponds to the situation when extrema of $\mathcal{F}$ fall on zero.

$$\begin{cases} \mathcal{F}(\mu_E) = 0 \\ \mathcal{F}'(\mu_E) = 0 \end{cases} \tag{S10}$$

Where $\mathcal{F}'$ denotes the derivative of $\mathcal{F}$ with respect to μ_E. This condition corresponds to changes in the number of solutions. The parameters comprised between these boundaries have the same number of solutions. This approach can be used to delimit the range of bistability or absence of solutions (as shown in Fig 5). Within such a region, the number of network states is obtained by determining the number of zero crossings of $\mathcal{F}$.

Parity of solutions

The number of zero crossings of the $\mathcal{F}$ function can be studied through its limits. Assuming that the F-I curves are supralinear($n_E > 1$ and $n_I > 1$), we get:

$$\begin{aligned} \lim_{\mu \to -\infty} \mathcal{F}(\mu) &= \infty \\ \lim_{\mu \to +\infty} \mathcal{F}(\mu) &= \lim_{\mu \to +\infty} a_E J_{EE} \mu_E^{n_E} - a_I J_{EI} \left(\frac{a_E \det J}{J_{EI}} \mu_E^{n_E} \right)_+^{n_I}. \end{aligned}$$

If $\det J > 0$, the second limit tends to $-\infty$. Therefore, the function has at least one zero and the $\mathcal{F}$ function for positive determinants has have an odd number of solutions (Mean-value theorem). On the other hand, if the determinant is negative, the second limit tends to $-\infty$. In that case, there is no guarantee that the system has a fixed solution and the number of solutions is even. Multiple roots (Eq S10) are counted separately in this calculation.

References

1. Allen Institute for Brain Science. Synaptic Physiology Coarse Matrix dataset; 2019. Available from: https://brain-map.org/explore/connectivity/synaptic-physiology.

2. Ko H, Hofer SB, Pichler B, Buchanan KA, Sjöström PJ, Mrsic-Flogel TD. Functional specificity of local synaptic connections in neocortical networks. Nature. 2011;473(7345):87–91.

3. Hofer SB, Ko H, Pichler B, Vogelstein J, Ros H, Zeng H, et al. Differential Connectivity and Response Dynamics of Excitatory and Inhibitory Neurons in Visual Cortex. Nature Neuroscience. 2011;14(8):1045–52.

4. Cossell L, Iacaruso MF, Muir DR, Houlton R, Sader EN, Ko H, et al. Functional organization of excitatory synaptic strength in primary visual cortex. Nature. 2015;518(7539):399–403.

5. Pfeffer CK, Xue M, He M, Huang ZJ, Scanziani M. Inhibition of inhibition in visual cortex: The logic of connections between molecularly distinct interneurons. Nature Neuroscience. 2013;16(8):1068–1076.

6. Khan AG, Poort J, Chadwick A, Blot A, Sahani M, Mrsic-Flogel TD, et al. Distinct learning-induced changes in stimulus selectivity and interactions of GABAergic interneuron classes in visual cortex. Nature Neuroscience. 2018;21(6):851–859.

7. Arkhipov A, Gouwens NW, Billeh YN, Gratiy S, Iyer R, Wei Z, et al. Visual physiology of the layer 4 cortical circuit in silico. PLoS Computational Biology. 2018;14(11):e1006535.

8. Billeh YN, Cai B, Gratiy SL, Dai K, Iyer R, Gouwens NW, et al. Systematic Integration of Structural and Functional Data into Multi-Scale Models of Mouse Primary Visual Cortex. Neuron. 2020 May;106(3):388–403.e18.

9. Priebe NJ, Mechler F, Carandini M, Ferster D. The contribution of spike threshold to the dichotomy of cortical simple and complex cells. Nature Neuroscience. 2004;7(10):1113–1122.

10. Tan AY, Brown BD, Scholl B, Mohanty D, Priebe NJ. Orientation selectivity of synaptic input to neurons in mouse and cat primary visual cortex. Journal of Neuroscience. 2011;31(34):12339–12350.

11. Alijani AK, Richardson MJ. Rate response of neurons subject to fast or frozen noise: from stochastic and homogeneous to deterministic and heterogeneous populations. Physical Review E. 2011;84(1):011919.

12. Fourcaud N, Brunel N. Dynamics of the firing probability of noisy integrate-and-fire neurons. Neural computation. 2002;14(9):2057–2110.

13. Van Hooser SD, Heimel JAF, Chung S, Nelson SB, Toth LJ. Orientation selectivity without orientation maps in visual cortex of a highly visual mammal. Journal of Neuroscience. 2005;25(1):19–28.

14. Woolsey TA, Van der Loos H. The structural organization of layer IV in the somatosensory region (SI) of mouse cerebral cortex: the description of a cortical field composed of discrete cytoarchitectonic units. Brain research. 1970;17(2):205–242.

15. Motta A, Berning M, Boergens KM, Staffler B, Beining M, Loomba S, et al. Dense connectomic reconstruction in layer 4 of the somatosensory cortex. Science. 2019;366(6469).

16. Meyer HS, Wimmer VC, Oberlaender M, De Kock CP, Sakmann B, Helmstaedter M. Number and laminar distribution of neurons in a thalamocortical projection column of rat vibrissal cortex. Cerebral cortex. 2010;20(10):2277–2286.

17. Hubel D, Wiesel T, LeVay S. Functional architecture of area 17 in normal and monocularly deprived macaque monkeys. In: Cold Spring Harbor Symposia on Quantitative Biology. vol. 40. Cold Spring Harbor Laboratory Press; 1976. p. 581–589.

18. Obermayer K, Blasdel GG. Geometry of orientation and ocular dominance columns in monkey striate cortex. Journal of Neuroscience. 1993;13(10):4114–4129.

19. Gur M, Snodderly DM. Physiological differences between neurons in layer 2 and layer 3 of primary visual cortex (V1) of alert macaque monkeys. The Journal of physiology. 2008;586(9):2293–2306.

20. O'Kusky J, Colonnier M. A laminar analysis of the number of neurons, glia, and synapses in the visual cortex (area 17) of adult macaque monkeys. Journal of Comparative Neurology. 1982;210(3):278–290.

21. Ramaswamy S, Courcol JD, Abdellah M, Adaszewski SR, Antille N, Arsever S, et al. The neocortical microcircuit collaboration portal: a resource for rat somatosensory cortex. Frontiers in neural circuits. 2015;9:44.

22. Markram H, Muller E, Ramaswamy S, Reimann MW, Abdellah M, Sanchez CA, et al. Reconstruction and Simulation of Neocortical Microcircuitry. Cell. 2015 Oct;163(2):456–492.

23. Seeman SC, Campagnola L, Davoudian PA, Hoggarth A, Hage TA, Bosma-Moody A, et al. Sparse recurrent excitatory connectivity in the microcircuit of the adult mouse and human cortex. Elife. 2018;7:e37349.

24. Keller D, Erö C, Markram H. Cell densities in the mouse brain: a systematic review. Frontiers in neuroanatomy. 2018;12:83.

25. van Vreeswijk C, Sompolinsky H. Chaotic balanced state in a model of cortical circuits. Neural computation. 1998;10(6):1321–71.

26. Rosenbaum R, Doiron B. Balanced networks of spiking neurons with spatially dependent recurrent connections. Physical Review X. 2014;4(2):021039.

27. Miller KD, Palmigiano A. Generalized paradoxical effects in excitatory/inhibitory networks. bioRxiv. 2020.

28. Ahmadian Y, Rubin DB, Miller KD. Analysis of the Stabilized Supralinear Network. Neural Computation. 2013 aug;25(8):1994–2037.

29. Tsodyks MV, Skaggs WE, Sejnowski TJ, McNaughton BL. Paradoxical effects of external modulation of inhibitory interneurons. Journal of neuroscience. 1997;17(11):4382–4388.

30. Wu YK, Zenke F. Nonlinear transient amplification in recurrent neural networks with short-term plasticity. Elife. 2021;10:e71263.

31. Kraynyukova N, Tchumatchenko T. Stabilized supralinear network can give rise to bistable, oscillatory, and persistent activity. Proceedings of the National Academy of Sciences. 2018;115(13):3464–3469.
