# Supplementary material for: Targeting operational regimes of interest in recurrent neural networks
Source: PLoS Comput Biol. 2023 May 15;19(5):e1011097. doi: 10.1371/journal.pcbi.1011097 (PMC10212141; doi:10.1371/journal.pcbi.1011097)
Supplement: Attachment — Submitted filename: PCOMPBIOL-D-22-01393_review.pdf [file pcbi.1011097.s004.pdf]

In the manuscript "Targeting operational regimes of interest in recurrent neural networks", the authors compare the fixed-point solutions of a firing rate equation to stationary states from a networks of LIF neurons. They do this by approximating the fI curve of the LIF neurons, which can be calculated analytically, by a simpler, power-law fI curve. They focus on several types of fixed-point solutions, including 1 – "Supersaturation", or a suppression of excitatory firing rates for increasing excitatory drive, 2 – Inhibition-stabilized sate, a state in which the E population would be unstable if left to its own devices and is stabilized by the I population, 3 – Bistable regimes or persistent activity.

The stated goal of the work, as put forth in the first sentence of the results section is "to understand how neural circuits comprising a few thousand neurons organize their spiking activity." This is a major goal of neuroscience in general, to put it lightly. This statement, together with the title itself of the manuscript, seem overly general. The authors have certainly not achieved this goal (nor could anyone in a single paper), so what, specifically, are the objectives of this manuscript? As I read through the paper I formulated several possible guesses as to their goals, based on the results presented, but was always left scratching my head as the authors never seemed to address the issues they themselves raised. At various points I thought the authors' main focus was on mean-field models, or finite-size effects, or contributing to the debate on balanced network vs. SSN. My main concern and criticism, would be that nothing here is truly novel. It may be that some technical findings, e.g. calculations or simulations, have indeed been done for the first time, but there is no novel methodological approach nor is there any conceptual novelty. Of course, I am open to the possibility of being wrong here, but then it is up to the authors to spell out in no uncertain terms precisely what they set out to do and why it is novel and interesting. I will be more specific in what follows.

1 – *Mapping between SSN and spiking activity*. There is, by now, a long history and extensive literature on mean-field models for neuronal activity. Most often the activity of spiking networks is approximated qualitatively through firing rate equations, but there are also many studies looking to make a quantitative, or pseudo-quantitative fit of mean-field models to spiking networks. The most well-known example is the work from Nicolas Brunel on LIF, including his work with Dani Amit, as well as his 2000 J Comp Neuro paper and the many papers which followed. This is a theory of fixed points and linear stability (also weakly nonlinear calculations from the 1999 paper with Vincent Hakim). The fixed point equations are actually given here as Eq.12. Solving these equations should lead to results which are quantitatively more accurate than those given here (if the authors use a network of LIF with delta synapses). These are the equations used in [49]. The authors approximate the fI curve from this theory using a threshold power-law function., which is reasonable approximation in a limited range of firing rates [32]. It should however, also be noted that there are other mean-field models out there, which fit the fixed-point structure (and even the dynamics) of spiking networks much better than the results presented here, such as the work of Tilo Schwalger (e.g. Schwalger et al PLoS Comp Biol 2017), or the exact mean-field model for QIF (Montbrió et al PRX 2015). None of this means the authors cannot fit their Eq.3 to a network of LIF neurons, but it does make statements such as "We establish a mapping between the SSN and spiking activity…" sound disingenuous, and statements such as "[I]t remains unclear how to translate computational results obtained with rate models to biologically plausible spiking networks" sound dismissive of the many contributions of other researchers. Nicolas Brunel derived a mean-field model for networks of LIF neurons. What the authors did was to fit a power law curve to the fI curve from that work, Eq.9. It also makes all of the fits from Figs.2-4 unsurprising. What is the novelty there? That a network of LIF neurons could operate in the

SSN regime? That has been shown. That the fit between theory and simulation is good? That has been shown. I'm not saying there is not a novel result here, it is just not clear to me.

2 – *Finite-size effects*. The authors begin by emphasizing the importance of system size, and, in fact, state that "Our goal is to understand how neural circuits comprising a *few thousand neurons* organize their spiking activity." Nonetheless, I do not see how this work is relevant for understanding the effect of system size on dynamics. Eq.3, which are an approximation to Eq.12, represent the mean-field of a network of LIF neurons in the thermodynamic limit, i.e. for infinite N. There is no N in the equations. The authors state that Eq.3 describes the activity in a network of 4000 neurons, but I disagree. They clearly have chosen the parameters in a network of 4000 neurons to match Eq.3, but could have done the same with 10,000, or 100,000, I guess. In Fig.6 they do scale the synaptic weights by N and show the effect on the mean activity. I would argue that this does not go far enough towards understanding how a network of "a few thousand neurons" organizes its activity, specifically, compared to a few hundreds or a few millions. Not enough to warrant stating it as a major goal of the paper. There is work, uncited here, on finite-size effects in spiking networks, dating back again to Brunel. These effects can be incorporated into the mean-field theory rigorously, e.g. Hakim and Brunel 1999. The work of Tilo Schwalger and also the collaboration between Michael Buice and Jack Cowan, specifically emphasize finite-size effects in their respective mean-field models. My personal guess is that the activity of too-small networks would just be too noisy and incoherent, while massively huge networks would be inefficient since the same job could be done with something smaller. If there is such a thing as a minimal functional circuit size, maybe it has to do with this trade-off. It's an interesting question.

3 – *Firing rates between 0-10Hz*. I found the authors' self-imposed constraint of maximal firing rate of 10Hz hard to understand. There is simply an overwhelming number of recordings of cells from many cortical areas with firing rates above 10Hz, including from papers which the authors cite as evidence that firing rates are always below 10Hz! Firing rate distributions, wherever quantified in cortex, tend to be lognormal-like, with most density near low rates, but also with a tail at higher rates. This arises in networks by virtue of passing a Gaussian input distribution through a nonlinear (near power-law) fI curve near threshold [32]. By the way, in that same paper [32] the authors fit the LIF network, using Brunel's theory, to the distribution of firing rates from somatosensory cortex (O'Connor et al Neuron 2010) where a significant fraction of cells fire above 10Hz! Also, look at the activity of cells in v1 from Finn, Priebe and Ferster, Neuron 2007, Fig.6A. The fI curve is power-law for rates up to 100Hz!

Other more specific comments:

4 – Introduction, 3rd paragraph: Regarding EI balance, the authors cite [17] but may want to look at Okun and Lampl, Nat. Neurosci. 2008.

5 – "slow timescales that are larger than the neuronal or synaptic variables are relevant fot eh analysis of biological circuits", followed by "Therefore, in the following we will consider firing rate dynamics which evolve on the time scales of a few tens of milliseconds to a hundred milliseconds which is slower than the *intrinsic times [sic] scales of the network*" (my italics). This is the reasoning used to set the temporal derivative to zero in Eq.2 and study the fixed point equations Eq.3. But if Eq.2 is meant to describe the network activity, what is the "intrinsic time scale of the network" if it isn't the time constants in Eq.2? Is there another set of equations, evolving on a longer time scale, which you are ignoring here? Where is that other

time scale coming from? I think it is ok to just say that you will be studying the fixed point equations, and not make it sound like you are studying the dynamics if you are not.

6 – Page 6: “Overall, we show that the mapping between SSN and spiking networks makes it possible…such that its activity follows a user-defined complex target dynamical trajectory”, referring to Fig.2E-F. Can’t you do this with any network? You are just forcing the rates to assume specific values by driving them with a given external input. It sounds trivial to me. Maybe I haven’t understood properly.

7 – In Fig.4B the authors vary the excitatory membrane time constant and in doing so vary the region of bistability. As far as I can tell, there is no membrane time constant in the mean-field equations Eq.3. So it should not affect the fixed point structure. So what parameter are they varying in Eq.3?
