# Supplementary material for: Targeting operational regimes of interest in recurrent neural networks
Source: PLoS Comput Biol. 2023 May 15;19(5):e1011097. doi: 10.1371/journal.pcbi.1011097 (PMC10212141; doi:10.1371/journal.pcbi.1011097)
Supplement: Attachment — Submitted filename: Reply_Pierre_SSN_Spiking.pdf [file pcbi.1011097.s005.pdf]

Dear Prof. Rubin, dear Prof. Marinazzo, dear reviewers,

We are grateful to all our three reviewers for their positive feedback, appreciation of our work and especially for their valuable, constructive comments which have greatly improved the accessibility of our manuscript.

Reviewer 1 suggested to improve the choice of wording and to better highlight the novelty and progress done in the present work to make it more explicit. We addressed this important comment and highlight the corresponding modifications in the manuscript, in our point-by-point responses and also consulted with the authors of the seminal papers mentioned by reviewer 1 to further support the presentation and discussion of our results.

Reviewer 2 (Tilo Schwalger) had many positive and helpful comments and mentioned "I found the manuscript interesting, sound and well-written. The theoretical approach via the SSN model is quite elegant. Furthermore, a theoretical understanding of the nonlinear response properties and neural computations in terms of biologically plausible spiking neural network model is an important current topic. It should also be noted that the model parameters (e.g. connectivity) have been chosen according to experimentally measured values from publicly available data." We have carefully implemented all comments of reviewer 2.

Reviewer 3 (Robert Rosenbaum) also had many positive and helpful comments and wrote: "[The authors] make several useful observations about the behavior and dynamics of spiking networks in various regimes. The manuscript is well-written and contributes some useful insight and methods. I only have a handful of comments that should be addressed." We have carefully implemented all comments of reviewer 3 into the revised manuscript.

In **bold** are the comments made by the reviewers, followed by our point-by-point responses with an indication of the corresponding changes in the manuscript.

Reviewer Comments & Author responses

Reviewer1:

In the manuscript "Targeting operational regimes of interest in recurrent neural networks", the authors compare the fixed-point solutions of a firing rate equation to stationary states from a networks of LIF neurons. They do this by approximating the fI curve of the LIF neurons, which can be calculated analytically, by a simpler, power-law fI curve. They focus on several types of fixed-point solutions, including 1 – "Supersaturation", or a suppression of excitatory firing rates for increasing excitatory drive, 2 – Inhibition-stabilized sate, a state in which the E population would be unstable if left to its own devices and is stabilized by the I population, 3 – Bistable regimes or persistent activity. The stated goal of the work, as put forth in the first sentence of the results section is "to understand how neural circuits comprising a few thousand neurons organize their spiking activity." This is a major goal of neuroscience in general, to put it lightly. This statement, together with the

title itself of the manuscript, seem overly general. The authors have certainly not achieved this goal (nor could anyone in a single paper), so what, specifically, are the objectives of this manuscript? As I read through the paper I formulated several possible guesses as to their goals, based on the results presented, [...]

Thank you for the suggestion to better highlight the novelty of our results relative to the state-of-the-art mean-field theories. We addressed this point via three steps. First, we have enhanced the clarity of the results by explicitly highlighting which novel insights our approach provides in comparison to previous theories. Second, we have carefully checked the literature mentioned by referee 1 and discussed its relation to the present work. Third, we have consulted with an author of the seminal mean-field models, Prof. Vincent Hakim, which according to the comments of referee 1 are most similar to our work. He confirmed that our approach is novel and provides an interesting perspective on the unsolved problem of predicting computational regimes of a spiking network model. Below, we provide a point-by-point reply to all comments of referee 1.

Major comments

1 – Mapping between SSN and spiking activity. There is, by now, a long history and extensive literature on mean-field models for neuronal activity. Most often the activity of spiking networks is approximated qualitatively through firing rate equations, but there are also many studies looking to make a quantitative, or pseudo-quantitative fit of mean-field models to spiking networks. The most well-known example is the work from Nicolas Brunel on LIF, including his work with Daniel Amit, as well as his 2000 J Comp Neuro paper and the many papers which followed. This is a theory of fixed points and linear stability (also weakly nonlinear calculations from the 1999 paper with Vincent Hakim). The fixed point equations are actually given here as Eq.12. Solving these equations should lead to results which are quantitatively more accurate than those given here (if the authors use a network of LIF with delta synapses). These are the equations used in [49]. The authors approximate the fI curve from this theory using a threshold power-law function., which is reasonable approximation in a limited range of firing rates [32]. It should however, also be noted that there are other mean-field models out there, which fit the fixed-point structure (and even the dynamics) of spiking networks much better than the results presented here, such as the work of Tilo Schwalger (e.g. Schwalger et al PLoS Comp Biol 2017), or the exact mean-field model for QIF (Montbrió et al PRX 2015). None of this means the authors cannot fit their Eq.3 to a network of LIF neurons, but it does make statements such as "We establish a mapping between the SSN and spiking activity..." sound disingenuous, and statements such as "[I]t remains unclear how to translate computational results obtained with rate models to biologically plausible spiking networks" sound dismissive of the many contributions of other researchers. Nicolas Brunel derived

a mean-field model for networks of LIF neurons. What the authors did was to fit a power law curve to the fI curve from that work, Eq.9. It also makes all of the fits from Figs.2-4 unsurprising. What is the novelty there? That a network of LIF neurons could operate in the SSN regime? That has been shown. That the fit between theory and simulation is good? That has been shown. I'm not saying there is not a novel result here, it is just not clear to me.

Thank you for suggesting to clarify our terminology and our goals. Our goal was to show that it is possible to predict the activity regime of a specific spiking network based on its connectivity configuration using a mathematically tractable mean field approximation of the network. To confirm that the problem of predicting computational regimes of a spiking network remains unsolved we have also consulted with several experts in mean-field and finite-size theories. These included Vincent Hakim, the co-author of the seminal studies that referee 1 cites, and Tilo Schwalger, one of the referees of the present manuscript and an expert on finite-size theories cited by referee 1. These experts agreed that our SSN model-based approach constitutes a novel and innovative method that advances our understanding on how recurrent spiking networks organize their activity in two ways. First, our Eq.3 precisely captures the mean population activity within a spiking neuronal network of 4000 neurons as well as 500.000 neurons (see Fig. 6). We can use the SSN to predict the expected operational regime of a spiking network (e.g., ISN regime, bistability or their absence) for a given size N, probability of recurrent connections p_{XY} and strength of recurrent connections j_{XY} (Eq.13), see Fig.5. Interestingly, it is also possible to use the SSN equations to invert the problem and design spiking networks with particular properties (e.g., ISN with or without E-I balance). Vincent Hakim and Tilo Schwalger confirmed that this was not possible before because previous self-consistent rate equations could not be solved analytically (including seminal work by Brunel and Hakim cited by referee 1). Second, both experts agreed that the degree of precision and mathematical tractability afforded by our mean field SSN equations (Eq. 3) is impressive and allows us to predict the location of many biologically interesting regimes, such as the ISN regime, supersaturation, and bistability in the spiking network parameter space. The precise understanding of the different activity regimes of the SSN model and whether they overlap to the degree shown in Fig. 5 was not previously done in any other self-consistent mean-field equations (including Brunel and Hakim [6]) and has not been derived until now.

Previous fundamental theoretical publications addressed the problem of deriving mean-field equations for the spiking network models. We cited [3, 5] in the first version of the manuscript, and we thank reviewer 1 for pointing to the work [6, 18, 24], which we now cite in our revised manuscript. We now discuss the importance of these mean-field models in more detail in the manuscript. Notably, these mean-field models could not predict the computational regime of a given spiking network with a particular number of neurons and connectivity configuration. Due to the complex implicit form of these models, a numerical approach was required to study them, whereas the SSN can be studied analytically. In the present work, we build a quantitative mapping between LIF spiking networks and the SSN rate model for which, despite its nonlinearity, many theoretical parameter analyses relating the network's computational regime to the connectivity configuration are available [1, 16]. For example, from the previous theoretical analysis of the SSN, we know that the relation between four parameters - J_{EI}, J_{II}, and the two

external inputs - determine whether an E/I network is in a supersaturation regime or not [1]. Such theoretical results provide a deeper understanding of network mechanisms than numerical simulations can. For instance, in the case of supersaturation, the numerical manipulations of irrelevant parameters (i.e., J_{EE} or the power law exponent n) would not switch the network to or from the supersaturation regime. Such precise understanding of the role of specific parameters for the presence or absence of a particular activity regime in spiking networks was not possible in previous mean field models to the best of our knowledge. Since the SSN model can accurately predict the computational regime of a network based on its connectivity configuration, we decided to analyze whether the predictions from the SSN model also translate to spiking neural networks and whether the computational regimes of a spiking network can be understood using the SSN model. The novelty of our work is that it provides an easy-to-implement numerical approximation of the spiking network through the SSN model, which is complementary to the previous mean-field theories and translates SSN-derived results to spiking neural networks. To emphasize the novelty of the presented results precisely and unambiguously, we added in the last paragraph of the introduction:
"With this work, we provide an easy-to-implement analytical approximation of the spiking network which relies on the SSN model. This allows us to predict the computational regime of a network of LIF neurons based on its parameters. "
Similarly we added in the end of the discussion:
" [...] our approach expands on these results by providing an alternative framework [...] through the power-law approximation. [...] Furthermore, the power-law function is designed to be both an accurate approximation of LIF dynamics and a simple mathematical expression. Thanks to this property, we showed that the SSN model can accurately match the activity of biologically realistic networks of LIF neurons including possible nonlinear features with tractable equations. This framework is useful to develop theoretical results which provide a deeper understanding of network mechanisms than numerical simulations [...]"

2 – Finite-size effects. The authors begin by emphasizing the importance of system size, and, in fact, state that "Our goal is to understand how neural circuits comprising a few thousand neurons organize their spiking activity." Nonetheless, I do not see how this work is relevant for understanding the effect of system size on dynamics. Eq.3, which are an approximation to Eq.12, represent the mean-field of a network of LIF neurons in the thermodynamic limit, i.e. for infinite N. There is no N in the equations. The authors state that Eq.3 describes the activity in a network of 4000 neurons, but I disagree. They clearly have chosen the parameters in a network of 4000 neurons to match Eq.3, but could have done the same with 10,000, or 100,000, I guess. In Fig.6 they do scale the synaptic weights by N and show the effect on the mean activity. I would argue that this does not go far enough towards understanding how a network of "a few thousand neurons" organizes its activity, specifically, compared to a few hundreds or a few millions. Not enough to warrant stating it as a major goal of the paper. There is work, uncited here, on finite-size effects in spiking networks, dating back again to Brunel. These effects can be incorporated into the mean-field theory rigorously, e.g. Hakim and Brunel 1999. The work of Tilo Schwalger and also the collaboration between Michael Buice and

Jack Cowan, specifically emphasize finite-size effects in their respective mean-field models. My personal guess is that the activity of too-small networks would just be too noisy and incoherent, while massively huge networks would be inefficient since the same job could be done with something smaller. If there is such a thing as a minimal functional circuit size, maybe it has to do with this trade-off. It's an interesting question.

Thank you for suggesting to clarify this important point. We do not study finite-size effects such as fluctuations around the mean since we use the mean-field-based SSN model and are careful not to use the term "finite-size" throughout the manuscript to avoid mix-ups. The confusion mentioned by referee 1 may come from the comparison we make to the balanced state, which explicitly considers infinitely large networks. Because of this, it require a tight balance of mean E and I currents to maintain a finite total current which would otherwise grow with N as $O(\sqrt{N})$. Our discussion on network size is meant to compare our mean-field approach to the balanced state model, which constitutes the limit for infinite N. Regarding the question of the network size (N), in the mean-field limit captured by Eq 3, the recurrent weights J_{XY} depend on the network size N because they can be decomposed into a product of single-synapse connection strength j_{XY}, the probability of connection p_{XY} and the number of neurons in each population N_Y (with $N = N_E + N_I$), see Eq 13. Our model covers all network sizes which have a well-defined mean population activity represented by the variable ν_X. It is correct that too low values of N would be noisy and incoherent due to finite-size effects. On the other hand, we have observed that networks with $N = 4000$ already have a well-defined mean firing rate activity which is amenable to a mean-field description. Furthermore, we can use our SSN framework for much larger N (which we explicitly do in Fig 6, with N ranging to 500 000 neurons) while the balanced-state limit is still not reached. Overall, there are many different mean field theories across the literature; each theory chooses specific quantities to stay constant as the network grows in size, while others will vanish in the limit of large networks. It is not a priori clear which of these theories, with their different limiting cases, is to be accurate for a particular spiking network with a specific number of neurons, synaptic current amplitudes etc. In our mean-field approach, we consider J_{XY} to be a finite quantity across all sizes of networks. Considering this mean-field model, we find that the network size we study (4000) is in a range where finite-size effects do not significantly contribute to the mean activity (i.e., the finite-size fluctuations are small enough such that the LIF network simulations match the model prediction well). At the same time, convergence to the balanced-state prediction is not achieved either. Since the network size we study is motivated by neuroanatomical data (See Supplement - Deriving biologically plausible neural network size for circuit simulations), our findings are relevant for our understanding of biologically reported recurrent circuits, e.g., in V1.

Tilo Schwalger, reviewer 2 and one of the experts in the field of finite-size models for recurrent circuits, indicated that our networks and the corresponding models could be best described by the term "biologically sized" rather than "medium-sized" to avoid confusion with the classic finite-size theories which consider mesoscopic effects. We have followed this advice throughout the manuscript. Besides, we added references to the publications mentioned by referee 1 studying finite-size effects in the introduction. Finally, in the discussion, we mentioned the study of

finite-size effects as a potential future development:
"[...] future work could analyze the dynamical properties of spiking networks (τ_{P_E} and τ_{P_I} in Eq. 2) to study such activity regimes as oscillations observed in the SSN model [16] and characterize the dynamical stability of network states or expand the model to characterize finite-size effects which become substantial in smaller networks [6, 7, 24]."

3 – Firing rates between 0-10Hz. I found the authors' self-imposed constraint of maximal firing rate of 10Hz hard to understand. There is simply an overwhelming number of recordings of cells from many cortical areas with firing rates above 10Hz, including from papers which the authors cite as evidence that firing rates are always below 10Hz! Firing rate distributions, wherever quantified in cortex, tend to be lognormal-like, with most density near low rates, but also with a tail at higher rates. This arises in networks by virtue of passing a Gaussian input distribution through a nonlinear (near power-law) fI curve near threshold [32]. By the way, in that same paper [32] the authors fit the LIF network, using Brunel's theory, to the distribution of firing rates from somatosensory cortex (O'Connor et al Neuron 2010) where a significant fraction of cells fire above 10Hz! Also, look at the activity of cells in v1 from Finn, Priebe and Ferster, Neuron 2007, Fig.6A. The fI curve is power-law for rates up to 100Hz!

Thank you for the suggestion to clarify the regime of firing rates in which our model operates. In our work, we do not consider transient activity as we assume the network operates at equilibrium (see "other more specific comments, point 5"). Similarly, we assume that the E and I populations are homogeneous (see Methods), meaning the firing activity we consider has to be the population average firing rate. While firing rates significantly over 10Hz in biological circuits do exist, they correspond to either short-lived dynamic responses or are only observed in a handful of cells and cannot be generalized to the whole population. Despite this, we find that low firing rates around 0-10Hz align with the experimentally reported values for sustained activity at the population level.

Let us review the references in our manuscript to support our focus on the 0-10 Hz firing rate range.

- Ref [33] provides data from patch clamp recordings of cells in rat somatosensory cortex and olfactory bulb. In the somatosensory cortex, the measured firing rate varies between cell-attached and whole-cell recordings, but the spontaneous activity was well below 1 Hz for regular spiking cells (Fig 5A), and up to 3.5 Hz for putative inhibitory cells. The evoked activity is obtained with a 50 ms air puff, which is not suitable to define an equilibrium firing rate (reported less than 1 spike/stimulus on average). Similarly, the olfactory bulb recording reported average firing rates of 0.6 Hz.
- Ref [34] studies the response of layer 5 pyramidal neurons in the visual cortex of awake mice to grating stimuli. Fig 4C shows the firing rate as a function of stimulus orientation. The mean firing rates are in the 0-2Hz range.

- Ref [35] studies the activity of V1 in freely behaving rats. Cell-specific recordings show some variability and some neurons can reach values higher than 10 Hz. However, the average firing rates are strictly in the 0-10 Hz range (Fig 1E).

- Ref [36] studies the barrel cortex of awake behaving mice. The mean firing rates for each population are below 10Hz during quiet wakefulness (Fig1B). The firing rates are also measured during passive sensory stimulation (Fig 2C), and can reach much higher values (reaching 50 Hz for FS neurons), but this is clearly just a transient response to the whisker deflection and the firing rates drop back after a few tens of ms. Finally, for active whisking, the reported average firing rates for each population are below 10 Hz before whisker-object contact and only FS neurons report higher than 10H z mean activity (12.5 Hz) after whisker contact. Again, note that these rates are only measured over a 50 ms time window.

- Ref [37] provides recorded activity from the somatosensory cortex of idle rats. This activity alternates between "Down" periods with close to 0 Hz firing and "Up" states where the firing rates are in the 0-6Hz range (Fig 1C). The duration of these periods is around 500 ms.

- Ref [38] provides data from whole-cell recordings of mouse V1 in response to grating stimuli. Fig 1A shows the response of three cells with different degrees of orientation selectivity, with firing rates in the 0-6Hz range. Each stimulus was presented for 1.5 s.

- Ref [39] provides a theoretical analysis of the distribution of firing rates observed in vivo. In particular, Fig 7 provides comparisons of LIF network simulations to recorded data from experimental papers. The first one (Hromádka et al., 2008) is the spontaneous activity in rat auditory cortex. While some cells fire at high rates, the mean and median activity of the population remains low (4.9 Hz and 2.8 Hz, respectively). The evoked activity at the population level is also measured and is of the same order (5.4-7 Hz mean and 2-2.7 Hz median). The second panel in [39] uses data from the mouse barrel cortex during active somatosensation (O'Connor et al Neuron 2010). Similarly, a few cells have a much higher firing rate than the rest, but the average rate across the whole barrel is only 7.4 Hz. A finer subdivision into layers shows that the firing rates in layers 2/3 and 6 are around 6 Hz, while the layers 4 and 5 have a higher activity, around 12 Hz.

- Ref [40] analyzes the response of pyramidal and PV neurons in mouse V1 layer 2/3. The evoked activity is shown in Fig 1D. The activity of pyramidal neurons is below 9.1 Hz while the PV mean firing rate is at 12 Hz. The mean spontaneous PV activity is at 2.1 Hz.

- Finally, we discuss the Figure 6A in Finn et al, 2007 mentioned by referee 1. The power law relationship between membrane potential and firing rate indeed holds for very high rates (>100Hz) but that still does not provide information about steady-state population-average firing rates. Here, the firing rates are measured over 30 ms bins in order to detect the maximum firing rate from a dynamic response to cycles of grating stimuli. The voltage traces in Fig 2A-C show that the network is far from equilibrium and the PSTH in Fig 8B,H show a high variability in the measured firing rate from one bin to the next. The reported instantaneous firing rates which can reach above 100Hz cannot be confused

with a sustained activity which a neuron could maintain over hundreds of ms in response to a constant stimulation.

In conclusion, our self-imposed range of 0-10Hz for the average population firing is consistent with many experimental reports. The reason we consider the upper bound of 10 Hz and not 12 or 8.4 Hz is because 10 is a round number and provides an obvious order of magnitude. To avoid that our readers interpret 10 Hz as strict upper limit for the average population firing we added the following explanation in the first paragraph of the results section:
"Neurons belong to one of two homogeneous populations, Excitatory (E) or Inhibitory (I). We restrict our analysis to the the range of 0-10 Hz, which is consistent with sustained population-averaged activity levels reported *in vivo* [17, 19, 14, 12, 15, 25, 21, 4]."

Finally, it is worth pointing out that the reason we impose a range of activity in which we operate is to bound the region of the Φ curve over which we fit a power law. Since Φ is not exactly a power law, changing the region of the curve we choose to approximate would lead to a change in resulting a, b, and n parameters. We could have performed the same analyses using a broader range (for example, 0-30 Hz). The only difference would be that the SSN approximation would match the LIF simulations marginally less precisely.

Other more specific comments:

4 – Introduction, 3rd paragraph: Regarding EI balance, the authors cite [17] but may want to look at Okun and Lampl, Nat. Neurosci. 2008.

Thank you for the suggestion. We added a reference to Okun and Lampl 2008, as well as to Rupprecht and Friedrich 2018.

5 – "slow timescales that are larger than the neuronal or synaptic variables are relevant fot eh analysis of biological circuits", followed by "Therefore, in the following we will consider firing rate dynamics which evolve on the time scales of a few tens of milliseconds to a hundred milliseconds which is slower than the intrinsic times [sic] scales of the network" (my italics). This is the reasoning used to set the temporal derivative to zero in Eq.2 and study the fixed point equations Eq.3. But if Eq.2 is meant to describe the network activity, what is the "intrinsic time scale of the network" if it isn't the time constants in Eq.2? Is there another set of equations, evolving on a longer time scale, which you are ignoring here? Where is that other time scale coming from? I think it is ok to just say that you will be studying the fixed point equations, and not make it sound like you are studying the dynamics if you are not.

Thank you for pointing this out. The intrinsic timescales are indeed the ones in Eq 2. The longer timescales observed in vivo which are not contained in this equation are the ones driving changes in the parameters of Eq.3. In particular, this can be the case of a dynamic trajectory of μ_{ext} which can occur as the stimulus presented to an animal changes in time. One could also

imagine changes in the excitability due to neuromodulation, or even adaptation. Overall, we are advocating that the study of neural networks at the steady state can be useful to analyze processes that unfold on slower timescales. This leads us to focus our attention on the fixed points of the SSN equation. Our aim is not to sound like we are studying dynamics, but to be explicit about the assumptions we make. We modified this section as follows to clarify our reasoning:
"*In vivo* cortical networks have been shown to respond to sudden stimulation with a transient (or *onset*) response that has a time scale of approximately 20 ms [13]. Meanwhile, *in vivo* recordings of multiple cortical areas have reported auto-correlation on a much slower timescales, of the order of hundreds of ms [8, 9, 10]. This suggests that the timescales over which biological networks typically evolve *in vivo* is driven by extrinsic factors like changes in feedforward input, rather than the intrinsic timescales τ_{P_X} which emerge from synaptic and neuronal variables. Therefore, in this work we restrict our analysis to the study of firing rates dynamics which evolve on sufficiently slow timescales to assume that the network operates at the equilibrium state. [...]"

6 – Page 6: "Overall, we show that the mapping between SSN and spiking networks makes it possible...such that its activity follows a user-defined complex target dynamical trajectory", referring to Fig.2E-F. Can't you do this with any network? You are just forcing the rates to assume specific values by driving them with a given external input. It sounds trivial to me. Maybe I haven't understood properly.

Fig 2E-F is a demonstration of the controllability a modeler can have over a spiking network thanks to the SSN. Full control of the desired firing level of both E and I populations is a key feature we set out to achieve with our approach. We do indeed force the rates to assume specific values by driving them with a given external input, but this can only be achieved because 1) the SSN model equations allow us to link rates to input and vice-versa, 2) we have ensured that the SSN tightly matches the spiking network simulation. The approximation provided by the SSN model is important because Φ is not tractable and it would be challenging to invert Eq.14 to determine the desired μ_{ext} for both E and I populations. Similarly, more simple rate models such as the threshold-linear model cannot match the LIF simulation accurately meaning that the resulting firing rate trajectory would not correspond to the target trajectory. As a side-note, this also ties with the previous question 5 regarding the dynamics. Since the whole firing rate trajectory emerges within 5s tightly following fast modulation of the network input, the simulation in Fig 2E,F shows that our steady state framework is still a valuable approximation even for the spiking network dynamics evolving on the time scale of a few hundred of milliseconds. To better explain the rational behind this, we added to the text relating to Fig 2E-F:
" It follows that the mapping between the steady states of the SSN and spiking neural networks provides a valuable approximation even for the slow spiking network dynamics."

7 – In Fig.4B the authors vary the excitatory membrane time constant and in doing so vary the region of bistability. As far as I can tell, there is no membrane time constant in the mean-field equations Eq.3. So it should not affect the fixed point structure. So what parameter are they varying in Eq.3?

The membrane time constant does not appear explicitly in Eq. 3, but the values of a,b, and n in Eq. 3 are obtained from the fit of the FI curve which does indeed vary with τ (see Fig 1C). The values of a,b and n corresponding to variations of τ_E in this figure are given in Table S4. To avoid confusion we added in the caption of Fig 4B:
"In the SSN, changes in τ_E correspond to changes in the parameters a_E, b_E and n_E."

Reviewer #2: The manuscript presents an analysis of the stationary firing rate responses of a excitatory-inhibitory network of noisy leaky integrate-and-fire neurons as a function of constant external inputs. The stationary firing rates are obtained as the steady-state solutions (fixed points) of the mean-field equations (similar to Brunel J. Comp. Neurosci. 2000). Importantly, the transfer functions of single neurons (”f-I curves”) that appear in the mean-field equations, are simplified by fitting a threshold-linear power-law function, which corresponds to the so-called stabilized supralinear network (SSN) model. The mapping to the SSN model enables a comprehensive mathematical analysis of the fixed points, and thus of the nonlinear firing rate responses to external stimuli, as a function of stimulus parameters and network connectivity. This analysis predicts a number of interesting and experimentally observed nonlinear responses (such as bistability, the paradoxical effect in inhibition stabilized networks and supersaturation), which may underlie important neural computations. The predicted responses are compared to simulations of the detailed spiking neural network and an excellent agreement is observed. Conversely, the paper can be seen as a demonstration that the highly tractable SSN model, for which many mathematical results exist, can be implemented with more realistic spiking neurons.

I found the manuscript interesting, sound and well-written. The theoretical approach via the SSN model is quite elegant. Furthermore, a theoretical understanding of the nonlinear response properties and neural computations in terms of biologically plausible spiking neural network model is an important current topic. It should also be noted that the model parameters (e.g. connectivity) have been chosen according to experimentally measured values from publicly available data. I have a few major concerns (see below), which should be relatively easy to address. Overall, I think that a revised manuscript that addresses all concerns listed below is suitable for publication in PLoS Computational Biology.

Major issues ==========

1. When the effect of network size is investigated, the authors ”re-scaled the recurrent connections J_{XY} by the factor $1/\sqrt{N}$ as a function of network size N”. I understand this sentence as follows: the weight at network size N is $J_N = r * J/\sqrt{N}$, where J is the weight before scaling (i.e. at $N = 4000$) and r is some fixed factor of proportionality (from Table S2, I conclude $r = \sqrt{4000}$). Now since $J_N = j * p * N$ (Eq.13), the actual weight per synapse in the LIF network is $j = (r * J/p) * N^{-3/2}$, i.e. the synaptic weights scale as $1/\sqrt{N^3}$ and not as $1/\sqrt{N}$ as in the limit for the balanced state theory. To obtain the desired balanced-state scaling ($j\ 1/\sqrt{N}$), the rescaled weights should instead be $J_N = J * \sqrt{N}/r$. If this latter scaling was indeed applied, then this is a minor issue and the authors should clarify the definition of the scaling more precisely using equations. Otherwise, Fig.6 and Fig.S2 should be redone with the correctly scaled weights (including new simulation) and the conclusions adapted if necessary. It may also explain why the authors did not observe a convergence to a balanced state in Fig.6. In this case, it would be interesting to know how large

this network needs to be such that the balanced-state theory becomes acceptable.

Thank you for spotting this typo, J and j were mixed up. It is indeed j_{XY} which is rescaled with $1/\sqrt{N}$ and not J_{XY}. We added the following clarification to avoid any confusion:
"we re-scaled the recurrent connections j_{XY} by the factor $1/\sqrt{N}$ as a function of network size N, and increased N from $N = 4 \times 10^3$ to 5×10^5 while keeping the connection probabilities fixed, leading to an effective re-scaling of the population-wide J_{XY} by $\sqrt{N}$."

2. A very similar analysis of the nonlinear response in LIF networks using the steady-state mean-field solutions has been recently performed by Sanzeni et al, Plos Comput Biol 2020 (ref.49). Given the similarity of the approach, the authors should clarify in more detail the difference and commonalities.

We agree that our discussion requires a more extensive comparison with the Sanzeni et al. 2020 results. The new paragraph follows the review of balanced state model extensions in the discussion section:
"Similarly, a recent article by Sanzeni et al. [23] used a different approach to study nonlinearities in spiking neural networks. The self-consistency solution was analyzed by using expansions of the Ricciardi Φ function in two limits where nonlinearities in the F-I curve occur: at response-onset, where firing rates are low and spiking is driven by noise, and at saturation where firing rates reach a maximum bounded by the refractory period of neurons. These two nonlinearities can lead to supersaturation and multisolution at the network level. While we do not consider saturation and refractory periods, our approach expands on these results by providing an alternative framework to describe the noise-induced nonlinearity through the power-law approximation. This power-law has a broader range of validity since it does not require to operate at the weak coupling limit. Furthermore, the power-law function is designed to be both an accurate approximation of LIF dynamics and a simple mathematical expression. Thanks to this property, we showed that the SSN model can accurately match the activity of biologically realistic networks of LIF neurons including possible nonlinear features with tractable equations and few parameters. This framework is useful to develop theoretical results which provide a deeper understanding of network mechanisms than numerical simulations. Nevertheless, it should be noted that the SSN, being a rate model, does not account for the recurrent noise originating from the activity in the network, which has been shown to make or break some nonlinear regimes [23]. Overall, the two approaches are complementary as the power-law framework focuses on mathematical tractability, matching LIF simulations and uses a unique activation function over the biological activity range while Sanzeni et al. [23] provide a finer analysis at two precise sources of nonlinearity."

In the following figure we illustrate the low firing rate firing rate approximation of the LIF F-I curve used by Sanzeni et al. compared to Φ and our power law fit. The transfer function used by Sanzeni et al. matches closely the Φ function for very low rates, but becomes quite inaccurate for higher levels of firing. Due to recurrent feedback loops, such deviations can get dramatically amplified in a network implementation. However, the power law is designed to approximate the Φ function over a given range, through the fitting procedure.

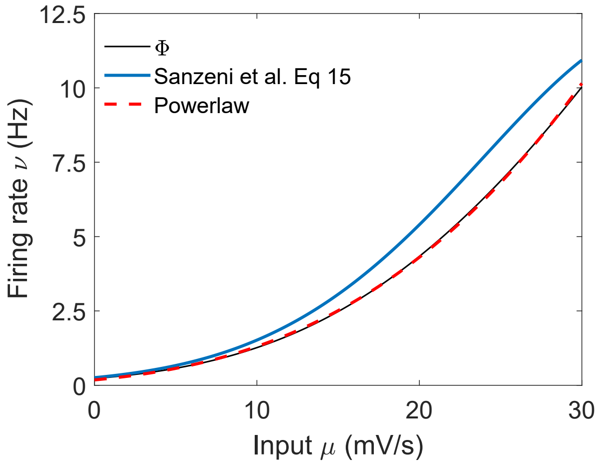

3. In many parts, it is stressed that the theory captures "medium-sized" spiking networks and the medium size appears to be a novel aspect of the paper. The "medium-size" theory is contrasted with the balanced-state theory, which is instead a "model for large or infinitely large networks". When reading the paper, I found the emphasis on medium size a little bit misleading because I expected a theory that captures finite size effects as in recent theoretical works. However, the present theory is just as well based on a mean-field limit of infinitely large networks as most other mean-field theories do, including the balanced-state theory; and as such it does not capture finite-size effects (e.g. fluctuation effects, correlations). Probably, the correct way to interpret their statement is as follows: there are different mean-field limits that lead to different limiting models and some are more useful than others in making predictions for the actual finite-size network at hand. Indeed, the SSN model is useful because the agreement with simulations of the medium-size LIF network is impressive, whereas the balanced-state theory fails to predict the responses of the medium-size network. The authors should make this rationale clear. They may also consider to replace "medium-sized" with the more innocent adjective "biologically-sized". Otherwise, the "medium-size" aspect could be confused with theoretical works on finite-size (or "mesoscopic") neural networks that deal specifically with the rather difficult problem of capturing finite-size effects such as fluctuations (e.g. Schwalger et al. Plos Comput Biol. 2017, Bressloff Phys Rev E 2010) or correlations (e.g. Tetzlaff et al. Plos Comput. Biol. 2012).

We agree that the term "medium-sized" can be misleading as it can imply that we aim to capture finite-size effects. Our approach focuses on the analysis of networks whose size allows us to neglect finite-size effects in a mean-field approximation, yet is far from the balanced limit. The term "biologically-sized" is indeed better suited to describe the network size we consider. All occurrences of "medium-sized" in the manuscript have been changed to "biologically-sized".

4. The balanced-state theory is also rejected based on the correlations between E and I input currents (Fig.2C, 4C, S2). The authors write, e.g. "If the network

operates at balance, the E and I recurrent inputs will cancel out and lead to a correlation which approaches -1. Here, the E-I correlation [...] is close to zero, demonstrating that the network operates far from E-I balance". I am not sure whether this statement about strong negative correlations in the balanced state is true (unfortunately, no reference is given), and thus whether the conclusions are correct. In the balanced network model, neurons become independent as N-> Infinity, and hence E-I current correlations should vanish, unless correlations are introduced externally (e.g. through shared external Poisson inputs as in Renart et al. Science 2010). However, in the present paper, there are no external correlations, and therefore the balanced state theory predicts 0 correlations (not -1), if I am not wrong. If I am correct, the authors should probably drop the corresponding figures.

We agree that the recurrent input correlation is not a good choice of measurement for balance. In the revised version of the manuscript, we decided to use a different measure of balance which we call the "balance factor", and which determines how much of the excitatory input is cancelled by inhibition. The balance factor is the ratio of the recurrent inhibitory current to the sum of recurrent excitation and feedforward inputs: $BF = \frac{\mu_{XI}}{\mu_{XE}+\mu_{ext}}$, so that in the case of perfect balance we have $BF = 1$. We used the balance factor to modify Fig 2C, 4C, S2. We also modified the captions and discussion of these figures in the manuscript.

We now write in section *Supersaturation*:
"To determine how close the network operates to E-I balance, we introduce the balance factor which measures the fraction of excitation that is cancelled by inhibition. We define the balance factor (BF) for population X, for positive external input μ_{ext}, as $BF = \mu_{XI}/(\mu_{XE} + \mu_{extX})$. If the network operates at balance, the recurrent inhibitory input will cancel out the total excitatory input and lead to a BF of 1. It should be clarified that the balance we are considering here must be understood in the sense of *tight balance* [26, 27, 20], meaning that inhibition matches the excitation and leads to near perfect cancellation. While a partial cancellation is considered a *loose balance* [22], it does not lead to characteristic features such as a predictable linear network response. Here, the BF measured at the peak E firing rate is close to 40% for E neurons and nearly null for I neurons, demonstrating that the network operates far from E-I balance (Fig2C)."

In the caption to Fig 2C we write:
"Boxplot of the balance factor for the E (blue) and I (red) neurons computed at the peak of E activity (see inset). The low values demonstrate that the network is operating far from E/I balance, as neurons in both populations receive significantly more excitation than inhibition. "

We write in section *Bistability* :
"Here again, the E-I balance is weak, as shown by the balance factor of inputs (Fig 4C), and the spiking activity is Poisson-like, as shown by the coefficient of variation of the ISIs Fig 4C. "

We write in the caption of Fig 4C:
"Balance factor for the E (blue) and I (red) neurons measured on the high activity branch (see inset). The BF values are far from 1, which indicates that the network is only loosely E/I

balanced."

We now write in section *Effect of network size on network response nonlinearity*:
"Next, we investigated whether these networks which are non-linear and do not conform to the balanced state prediction can have a balance factor close to 1. We have shown that the values of BF are far from 1 in the bistable network of 4000 neurons (Fig 4C), which is an indicator that the network is operating far from balance. However, we found that the balance factor of inputs can be network size dependent: increasing the size N from 4000 to 40000 strongly increased the inhibition to excitation ratio even though the firing rate activity in both cases does not conform to the balanced state solution (Fig S2). In summary, the observation of significant cancellation of incoming excitatory and inhibitory signals does not guarantee that the balanced state framework is applicable to predict the firing activity. Even small deviations from tight balance (BF <1) can lead to significant deviation in the resulting network activity regime."

We write in the caption of Fig S2:
"As the size of networks increases, the incoming excitatory and inhibitory inputs become more balanced. This is measured in E (blue) and I (red) neurons for the same bistability case as Fig 4 and Fig 6C, at the point where the excitatory firing rate reaches 10 Hz (see inset above, for each network size). The balance factor measures the fraction of the excitatory input that is compensated by inhibition, $\frac{\mu_{XI}}{\mu_{XE}+\mu_{ext}}$. For $N = 4000$ neurons, E and I neurons receive nearly twice as much excitation as inhibition (BF$\approx$ 55% and 43% respectively) whereas for $N = 40000$, most excitatory input is cancelled by inhibition (BF$\approx$ 92% and 86% respectively). This shows that E-I balance gets tighter as the network size increases even though the firing rates are far from the balanced state limit (dashed line, inset) and the network response remains non-linear at low input level."

Minor issues ==========

- p.3 "the F-I curve of a LIF neuron given by the Ricciardi transfer function Φ" and Fig.1: The Richiardi transfer function assumes Gaussian white noise in the voltage dynamics of the LIF model but this assumption is not mentioned in the main text (it is mentioned in SI, though).

We added this assumption in the manuscript:
"Our starting point is the observation that a power-law function can accurately describe the F-I curve of a single LIF neuron in response to white noise input across different membrane time constants τ and input noise values σ."

- Please provide the full expression for the input current I in Eq.8, including recurrent inputs and noise. Importantly, please provide equations for the noise in the main text, otherwise it is ambiguous what e.g. the parameter sigma is. In the case of Gaussian white noise, σ^2 cannot be its variance (as written below Eq.8) and sigma cannot be its standard deviation (as written in SI) because the variance of white noise is infinity. Maybe call it noise strength or infinitesimal variance.

We added the definition of the input I after the LIF equation (Eq 8) in the main manuscript, section Materials and Methods, where we introduced the parameters μ and σ. The breaking down of I into its recurrent and feedforward components is done in the section Self-consistency network solution, where the single-neuron transfer function is used to determine the activity of neurons in a recurrent network. We now refer to σ as "noise strength".

- Sec. "Noise contribution of exponential synapses" in SI is not very clear, esp. the derivation of the effective sigma for colored noise, which seems rather hacky. There are theoretical results on this problem, most importantly Alijani & Richardson (Phys Rev. E 2011), who have a nice rescaling of the Ricciardi function for colored noise (basically "take sigma of the effective model with Gaussian white noise such that the resulting var(V) equals the var(V) of the actual model with colored noise (both in absence of threshold). It will lead to similar firing rates for white and colored noise"). Another result on the firing rate for small τ_s has been derived by Brunel (Fourcaud-Trocme & Brunel Neural Comput 2002).

Thank you for spotting this and for the useful references. We agree that this section was confusing, it was related to a project direction we did not further pursue in our manuscript. We removed this section and added a paragraph in SI explaining why we used exponential synapses in our simulations:

"Finally, for some spiking network simulations we used exponential synapses with randomly distributed synaptic delays to avoid synchronization (section LIF spiking network simulation). In those cases, the values of the synaptic time constant τ_S and the delay D are included only to stabilize the network around the steady state. The particular parameter values should not substantially affect the mean-field solution as we operate at steady state. While the use of exponential synapses leads to changes in the properties of the recurrent noise [2, 11], the contribution of recurrent noise is negligible compared to external noise in our framework (section Mapping LIF network - SSN). The values of τ_S and D are provided in Table S3."

-p. 3 "the input to a neuron in a recurrent network [...] is equivalent to an Ornstein-Uhlenbeck process or white noise if the number of incoming PSPs is sufficiently large and the activity is irregular.": The condition of irregular activity is not precisely true. The white noise or OU process requires that the spiking activity is Poisson, i.e. it has no temporal correlations. Any stochastic spike train is, strictly speaking, irregular and bursty spiking is even more irregular than a Poisson process (CV, Fano factor > 1).

Indeed. We corrected the manuscript which now reads:
"the input to a neuron in a recurrent network, which is a superposition of postsynaptic potentials (PSPs), is equivalent to an Ornstein Uhlenbeck process or white noise if the number of incoming PSPs is sufficiently large and the activity is temporally uncorrelated or Poissonian."

- Terminology "E-I balance": E.g., p.5 "To determine how close the network op-

erates to E-I balance...": in this paper, the term "balance" is used in the rather narrow sense of the balanced-state theory of van Vreeswijk and co-workers. But E-I balance is more general, e.g. the model of Brunel (J. Comp Neurosci 2000) is also balanced if g=4 (perfect balance) or at least g>=4 and it has the same nonlinear response properties as in the present manuscript. Please provide a clear definition and terminology of "balance".

It is true that we use the term E-I balance in a strict sense, while it could apply to looser conditions. To avoid any confusion, we added a clarification in the paragraph on p.5 (as already mentioned in major issues point 4):
"It should be clarified that the balance we are considering here must be understood in the sense of *tight balance* [26, 27, 20], meaning that inhibition matches the excitation and leads to near perfect cancellation. While a partial cancellation is considered a *loose balance* [22], it does not lead to characteristic features such as a predictable linear network response."

- p.5 reference to Eq.18: Eq.18 seems not to be in the main paper and probably needs reference to "Supporting Information".

Thank you. The numbering of equations in SI has been corrected such that the numbers now start with S. The reference to Eq 18 now refers to Eq S6.

- p.5 "correlation which approaches -1": In which limit? Maybe you mean 'correlation close to -1'? But see my point 4 above.

Thank you. As mentioned above, we now use the balance factor (BF) as a measure of balance. This sentence is now replaced with:
"If the network operates at balance, the recurrent inhibitory input will cancel out the total excitatory input and lead to a BF of 1."

Typos: ====== -p.25 (SI), very last equation: equation for $\delta\dot{\nu}_I$ is wrong

Thanks. It is now corrected :
$\delta\dot{\nu}_E = f'(\mu_E^0)\delta\mu_E = f'(\mu_E^0)(J_{EE}\delta\nu_E - J_{EI}\delta\nu_I)$
$\delta\dot{\nu}_I = g'(\mu_I^0)\delta\mu_I = g'(\mu_I^0)(J_{IE}\delta\nu_E - J_{II}\delta\nu_I)$

-p.28 (SI): "for supersaturating networks $(JEI/r - JII)$": do you mean $(JII/r - JEI < 0)$?

Yes! Thank you. It is now corrected :
"In particular, for supersaturating networks $(J_{II}/r - J_{EI} < 0)$ for which $\det J$ is negative, $J_{IE}/r - J_{EE}$ is negative"

-p.12: misprint in last sentence of Results: $\rho_{E,I}$?-1

This expression has now been changed to $(BF$ <1).

-p.30: subscript "s" missing on τ_s in unnumbered equation

The section was removed.

-p.30: equation for covariance: absolute value sign of $|\Delta t|$ is missing

The section was removed.

Reviewer #3: In this manuscript, the authors use a least-squares fit to establish a mapping in firing rate space between E-I networks of LIF neuron models ("spiking networks") to two-dimensional rate models with power-law f-I curves ("SSN networks"). They verify that this mapping can predict firing rates and other dynamical properties of spiking networks using results from the considerably simpler SSN networks. In doing so, they make several useful observations about the the behavior and dynamics of spiking networks in various regimes.

The manuscript is well-written and contributes some useful insight and methods. I only have a handful of comments that should be addressed.

1) Abstract: "Neural computations emerge from recurrent neural circuits that comprise hundreds to a few thousand neurons" While it could be argued that "local" circuits in the cerebral cortex are restricted to approximately this size (even then, I'm not totally convinced, but I digress), the statement as written is too general. Please rephrase to clarify the context in which this statement is true (local circuits (or "a computational unit" eg column), cerebral cortex, rodent?).

We agree that it might be too general, especially as the very first sentence in the abstract. Therefore, we modified it to incorporate the suggested clarifications:
"Neural computations emerge from local recurrent neural circuits or computational units such as cortical columns that comprise hundreds to a few thousand neurons."

We also agree that the question of what defines a computational unit, a subnetwork or a canonical circuit is very complex and somewhat ill-defined. We dedicated a section in the supplement to discuss how neuroanatomical data from different brain regions and animal species can motivate our choice of a unit with 4000 neurons.

2) "(LIF) model ... which represents an accurate description of cortical neurons both in vivo and in vitro"

This is a strong statement that many in the field would disagree with. It should be softened. The LIF can certainly be described as a useful approximation to real neurons or something similar, but I do not think that most computational neuroscientists (even those who use LIFs in the work) would agree with the current phrasing.

It is true that the statement might be too strong, and we agree with the suggestion of replacing "accurate" with "useful".

3) Somewhere in the paragraph(s) following Eq (1): Please specify that a, b, and n were fit to LIF simulations. This would help the reader understand the approach.

Thank you for pointing this out. The a, b, and n parameters were fitted using the Ricciardi Φ function which characterizes the transfer function of LIF neurons. To help clarify this, we

modified Fig 1B to show Φ and modified the sentence after Eq 1:
"The constants a, b, and n, which are obtained by fitting the Φ function (Eq 9), characterize the power-law approximation with a scaling pre-factor a, an input threshold b upon which the neuron starts firing, and an exponent n."

4) In the paragraph following Eq (2): Give an equation for μ_X.

Thanks, we added the following text after Eq 2:
"Where ν_X, $X \in \{E, I\}$ are the firing rates of the two populations, the parameters a_X, b_X, and n_X are given by the F-I curve fit of E and I neurons (black crosses in Fig 1C), and μ_X is the total input to the neurons in each population. In a recurrent E-I network, μ_X is the sum of recurrent ($J_{XE}\nu_E - J_{XI}\nu_I$, see Eq 12) and feedforward (μ_{extX}) inputs to each population."

5) Before Eq (3): You explain that you will focus on the equilibrium states, but you are selling yourself short since you also consider the stability of such states. This is worth mentioning here to avoid giving the impression that stability will be ignored.

In the context of this work we do not analyze the stability of steady states in the SSN, but this analysis has been done in Kraynyukova and Tchumatchenko [16]. We do however mention necessary conditions for the stability in the balanced state (equation S4). The necessary and sufficient conditions for stability are not investigated further because they depend on the dynamics which are out of the scope of this work.

6) Page 11: The balanced state limit is discussed and plotted in the figures, but an equation for the rates in this limit is not given (unless I missed it?). Please write the equation here or in Methods.

Thank you for pointing this out. We now added a reference to the balanced firing rate equation in Eq S1 in the main text:
"By considering the feedforward input before scaling ($\mu_{ext}/\sqrt{N}$) [26, 27], the network response should converge toward a single linear solution - the balanced limit (Eq S1, dashed line in Fig 6D,E)."
and in the caption of Fig 6:
"(D) depicts the same network as in panel C but now without external inputs scaling to highlight the balanced solution. The balanced state predicts a linear solution for the $N \to \infty$ limit (Eq S1, dashed line)."

7) Page 11 "By considering the feedforward input before scaling": I assume what is going on here is that external input is multiplied by $\sqrt{N}$ in the simulations (consistent with the balanced net approach), so it needs to be scaled by 1/sqrt(N) to say that rates converge to a fixed value. I think that many readers will not follow this reasoning, especially since I do not see the original $\sqrt{N}$ scaling of external input mentioned anywhere (unless I missed it). Please clarify that this scaling is used (if it is) and make this statement more clear. Note that this explanation might come

naturally from writing an equation for rates in the balanced limit, as suggested above.

It is true that we mentioned the rescaling of recurrent synaptic weights but did not explicitly mention the rescaling of external inputs. We now added a clarification after the mention of the balanced state convention:
"In the balanced state convention, the feedforward input follows the same rescaling and grows with $\sqrt{N}$. We show in Fig 6A,C,E the network response to the effective feedforward input after scaling (μ_{ext}), and in Fig 6B,D,F the network response as a function of the external input before scaling, ($\mu_{ext}/\sqrt{N}$) to highlight a possible convergence to the balanced solution as N grows."

8) Last sentence before Discussion: There appears to be a LaTeX rendering error in the parenthesis. I see an upside down question mark in the pdf.

Thanks for spotting this typo, this expression has now been changed to (BF <1), where BF is the balanced factor which we introduced to replace the input current correlation ρ_{EI} as measure of how balanced the network is: $BF = \frac{\mu_{XI}}{\mu_{XE}+\mu_{extX}}$.

9) Methods LIF spiking network: The authors mentions the occurrence of synchronous spiking in some of their LIF simulations with delta synapses. While synchrony can be a real effect in such networks, it can also arise spuriously if the membrane potentials are updated immediately after each spike. Networks with exponential synapses do not have this problem. To avoid this problem in networks with delta synapses, the post-spike increments can be stored until after all membrane potentials have been updated (i.e., until after the forward Euler update if a forward Euler solver is used).

I do not know which approach the authors take, but if they increment membrane potentials immediately after each spike then they could consider modifying their code to perform post-spike increments after updating all of the membrane potentials to see if this modification avoids synchrony without resorting to exponential synapses.

Thank you for the suggestion. We checked in our spiking network simulation code and it appears this is already how it was implemented.

10) Methods LIF spiking network: Please give the values of time constant and delay parameters used for exponential synapses. Check that parameter values for all other parameters used in the study are also given somewhere.

Indeed, we forgot to provide the parameters used for exponential synapses. We also carefully checked that no other parameter was left out. We added a table providing the values of τ_S and D in the supplement, following Table S2, along with a short additional paragraph:
"Finally, for some spiking network simulations we used exponential synapses with randomly

distributed synaptic delays to avoid synchronization (section LIF spiking network simulation). In those cases, the values of the synaptic time constant τ_S and the delay D are included only to stabilize the network around the steady state. The particular parameter values should not substantially affect the mean-field solution as we operate at steady state. While the use of exponential synapses leads to changes in the properties of the recurrent noise [2, 11], the contribution of recurrent noise is negligible compared to external noise in our framework (section Mapping LIF network - SSN). The values of τ_S and D are provided in Table S3."

	τ_S	D	Affected Synapses
Fig. 4A-B	250	0 - 150	EE,EI,IE,II
Fig. 6C-D	200	0 - 1000	EE,II
Fig. 6E-F	200	0 - 500	EE,II

Table S3 : Synaptic parameters used in simulations with exponential synapses to prevent synchronization. The exponential synapse time constant τ_S and the synaptic delay D are given in ms. The synaptic delay for each synapse is drawn randomly from a uniform distribution in the given range. For all other LIF simulation and for synapses not listed as "Affected synapses", we used the default delta-synapses without delay.

References

[1] Ahmadian, Y., Rubin, D. B., and Miller, K. D. (2013). Analysis of the Stabilized Supralinear Network. *Neural Computation*, 25(8):1994–2037.

[2] Alijani, A. K. and Richardson, M. J. (2011). Rate response of neurons subject to fast or frozen noise: from stochastic and homogeneous to deterministic and heterogeneous populations. *Physical Review E*, 84(1):011919.

[3] Amit, D. J. and Brunel, N. (1997). Model of global spontaneous activity and local structured activity during delay periods in the cerebral cortex. *Cerebral cortex (New York, NY: 1991)*, 7(3):237–252.

[4] Atallah, B. V., Bruns, W., Carandini, M., and Scanziani, M. (2012). Parvalbumin-expressing interneurons linearly transform cortical responses to visual stimuli. *Neuron*, 73(1):159–170.

[5] Brunel, N. (2000). Dynamics of sparsely connected networks of excitatory and inhibitory spiking neurons. *Journal of computational neuroscience*, 8(3):183–208.

[6] Brunel, N. and Hakim, V. (1999). Fast global oscillations in networks of integrate-and-fire neurons with low firing rates. *Neural computation*, 11(7):1621–1671.

[7] Buice, M. A., Cowan, J. D., and Chow, C. C. (2010). Systematic fluctuation expansion for neural network activity equations. *Neural computation*, 22(2):377–426.

[8] Cavanagh, S. E., Hunt, L. T., and Kennerley, S. W. (2020). A diversity of intrinsic timescales underlie neural computations. *Frontiers in Neural Circuits*, 14:81.

[9] Cavanagh, S. E., Wallis, J. D., Kennerley, S. W., and Hunt, L. T. (2016). Autocorrelation structure at rest predicts value correlates of single neurons during reward-guided choice. *elife*, 5:e18937.

[10] Chen, G. and Gong, P. (2019). Computing by modulating spontaneous cortical activity patterns as a mechanism of active visual processing. *Nature communications*, 10(1):1–15.

[11] Fourcaud, N. and Brunel, N. (2002). Dynamics of the firing probability of noisy integrate-and-fire neurons. *Neural computation*, 14(9):2057–2110.

[12] Gentet, L. J., Kremer, Y., Taniguchi, H., Huang, Z. J., Staiger, J. F., and Petersen, C. C. (2012). Unique functional properties of somatostatin-expressing gabaergic neurons in mouse barrel cortex. *Nature neuroscience*, 15(4):607–612.

[13] Guest, J. M., Bast, A., Narayanan, R. T., and Oberlaender, M. (2021). Thalamus gates active dendritic computations in cortex during sensory processing. *bioRxiv*.

[14] Hengen, K. B., Lambo, M. E., Van Hooser, S. D., Katz, D. B., and Turrigiano, G. G. (2013). Firing rate homeostasis in visual cortex of freely behaving rodents. *Neuron*, 80(2):335–342.

[15] Jercog, D., Roxin, A., Bartho, P., Luczak, A., Compte, A., and de la Rocha, J. (2017). Up-down cortical dynamics reflect state transitions in a bistable network. *Elife*, 6:e22425.

[16] Kraynyukova, N. and Tchumatchenko, T. (2018). Stabilized supralinear network can give rise to bistable, oscillatory, and persistent activity. *Proceedings of the National Academy of Sciences*, 115(13):3464–3469.

[17] Margrie, T. W., Brecht, M., and Sakmann, B. (2002). In vivo, low-resistance, whole-cell recordings from neurons in the anaesthetized and awake mammalian brain. *Pflügers Archiv*, 444(4):491–498.

[18] Montbrió, E., Pazó, D., and Roxin, A. (2015). Macroscopic description for networks of spiking neurons. *Physical Review X*, 5(2):021028.

[19] Rancz, E. A., Franks, K. M., Schwarz, M. K., Pichler, B., Schaefer, A. T., and Margrie, T. W. (2011). Transfection via whole-cell recording in vivo: bridging single-cell physiology, genetics and connectomics. *Nature neuroscience*, 14(4):527–532.

[20] Renart, A., De La Rocha, J., Bartho, P., Hollender, L., Parga, N., Reyes, A., and Harris, K. D. (2010). The asynchronous state in cortical circuits. *science*, 327(5965):587–590.

[21] Roxin, A., Brunel, N., Hansel, D., Mongillo, G., and van Vreeswijk, C. (2011). On the distribution of firing rates in networks of cortical neurons. *Journal of Neuroscience*, 31(45):16217–16226.

[22] Rubin, D. B., Van Hooser, S. D., and Miller, K. D. (2015). The stabilized supralinear network: a unifying circuit motif underlying multi-input integration in sensory cortex. *Neuron*, 85(2):402–417.

[23] Sanzeni, A., Histed, M. H., and Brunel, N. (2020). Response nonlinearities in networks of spiking neurons. *PLoS computational biology*, 16(9):e1008165.

[24] Schwalger, T., Deger, M., and Gerstner, W. (2017). Towards a theory of cortical columns: From spiking neurons to interacting neural populations of finite size. *PLoS computational biology*, 13(4):e1005507.

[25] Tan, A. Y., Brown, B. D., Scholl, B., Mohanty, D., and Priebe, N. J. (2011). Orientation selectivity of synaptic input to neurons in mouse and cat primary visual cortex. *Journal of Neuroscience*, 31(34):12339–12350.

[26] van Vreeswijk, C. and Sompolinsky, H. (1996). Chaos in neuronal networks with balanced excitatory and inhibitory activity. *Science*, 274(5293):1724–1726.

[27] van Vreeswijk, C. and Sompolinsky, H. (1998). Chaotic balanced state in a model of cortical circuits. *Neural computation*, 10(6):1321–71.
