# Supplementary material for: Targeting operational regimes of interest in recurrent neural networks
Source: PLoS Comput Biol. 2023 May 15;19(5):e1011097. doi: 10.1371/journal.pcbi.1011097 (PMC10212141; doi:10.1371/journal.pcbi.1011097)
Supplement: Attachment — Submitted filename: Response_to_Reviewers_rev2.pdf [file pcbi.1011097.s006.pdf]

Dear Prof. Rubin, dear Prof. Marinazzo, dear reviewers,

We are very thankful to the three reviewers for their support and appreciation of our work.

Reviewers 1 and 2 are satisfied with our edits and replies to the issues they raised in the previous revision round.

Reviewer 3 is also satisfied with our response, but raises an extra point regarding the biological realism of the synaptic parameters we use to prevent synchronization in the network. In order to address this concern, we ran the relevant simulations again using more realistic parameters and edited the manuscript accordingly.

In **bold** are the comments made by the reviewers, followed by our responses with an indication of the corresponding changes in the manuscript.

Reviewer Comments & Author responses

Reviewer1:

I thank the authors for their thorough responses to my questions, comments and criticisms. While I still am not 100% on board with all of the claims made here, what sort of world would it be if everyone agreed on everything? I would be happy to see the article be published in PLoS Comp Biol.

Thank you for the support. And thank you again for helping us identify previous points of confusion.

Reviewer2:

The authors have addressed all my concerns satisfactory.

Thank you again for very insightful comments.

Reviewer3:

The authors addressed all of my concerns with only one minor issue remaining. In response to one of my previous comments, they added a table of synaptic time constants and delay times (Table S3). The reported time constants are 200-250ms whereas synaptic timescales in cortical synapses are closer to 5-10ms. Similarly, the delay timescales are several orders of magnitude larger than realistic values for the size of networks studied in the manuscript. An unmyelinated axon can conduct a signal across 1 meter in 100ms, but the authors use delays several times longer than 100ms. Since the networks modeled in the manuscript are likely smaller than 1mm, these are highly unrealistic delays.

If the simulations work well (e.g., stability is achieved) with more realistic parameter values, then the simulations should be replaced with more realistic ones.

If the authors needed to choose their biologically unrealistic timescales to achieve stability in their simulations, this is a weakness of their study. It does not preclude the publication of the manuscript in PCB. All studies have weaknesses and, more specifically, nearly all modeling studies use some unrealistic parameter values. But the choice of parameter values should be mentioned and explained somewhere in the Results and/or Discussion.

Thank you for directing our attention on this. It is clear that in the specific case of the synaptic parameters τ_S and D, we did not aim for biological realism. Since we only introduce these in order to prevent synchronization and they do not play a role in the mean field solution Eq.14, we did not give much attention to the specific value of these parameters, as long as they fulfilled their stabilizing purpose. This was definitely an oversight on our side, since more realistic parameter values could be used instead. Following your suggestion we ran the concerned simulations with synaptic time constant τ_S=10ms and synaptic delay D randomly drawn from the 0-100ms range. As it turns out, the network simulations are still asynchronous. The resulting firing rates are barely affected by the change of synaptic parameters. The small difference we observe can be explained by the effect of recurrent noise which was silenced with much longer time constants τ_S. Thank you for your suggestion which led us to cleaner results.

We updated the relevant figures in the manuscript. We modified Table S3 and its caption:

	τ_S	D	Affected Synapses
Fig.4<br>Fig.6C<br>Fig.6E<br>Fig.S2	10	0-100	EE,II

Table S3 : Synaptic parameters used to prevent synchronization. The exponential synapse time constant τ_S and the synaptic delay D are given in ms. The synaptic delay for each synapse is drawn randomly from a uniform distribution in the given range. The EI and IE synapses are not affected and use delta-synapses without delay. In all figures not listed here, the LIF simulations were performed with the default delta-synapses without delay.

Finally, we added a statement about how our synaptic delays can be unrealistically high in the Material and methods section:
"We use the exponential synapse in the spiking network simulation in Fig.4, Fig.6C, Fig.6E and Fig.S2, and delta synapses in all other cases. The exponential synapse parameters can be found in Table S3. It should be noted that the synaptic delay values we use are higher than experimentally reported [3, 1, 2], but this jitter only serves to prevent population synchrony and does not affect the steady state network activity."

References

[1] Barnes, S. J., Cheetham, C. E., Liu, Y., Bennett, S. H., Albieri, G., Jorstad, A. A., Knott, G. W., and Finnerty, G. T. (2015). Delayed and temporally imprecise neurotransmission in reorganizing cortical microcircuits. *Journal of Neuroscience*, 35(24):9024–9037.

[2] Katz, B. and Miledi, R. (1965). The measurement of synaptic delay, and the time course of acetylcholine release at the neuromuscular junction. *Proceedings of the Royal Society of London. Series B. Biological Sciences*, 161(985):483–495.

[3] Purves, D., Augustine, G., Fitzpatrick, D., Katz, L., LaMantia, A., McNamara, J., and Williams, S. (2001). Increased conduction velocity as a result of myelination. neuroscience.
